# Detecting a Surprisingly Low Transmission Distance in the Early Phase of the 2009 Influenza Pandemic

**DOI:** 10.1038/s41598-017-12415-2

**Published:** 2017-09-26

**Authors:** Valentina Marziano, Andrea Pugliese, Stefano Merler, Marco Ajelli

**Affiliations:** 1 Bruno Kessler Foundation, Trento, Italy; 2 Department of Mathematics, University of Trento, Trento, Italy; 3 Laboratory for the Modeling of Biological and Socio-technical Systems, Northeastern University, Boston, MA USA

## Abstract

The spread of the 2009 H1N1 influenza pandemic in England was characterized by two major waves of infections: the first one was highly spatially localized (mainly in the London area), while the second one spread homogeneously through the entire country. The reasons behind this complex spatiotemporal dynamics have yet to be clarified. In this study, we perform a Bayesian analysis of five models entailing different hypotheses on the possible determinants of the observed pattern. We find a consensus among all models in showing a surprisingly low transmission distance (defined as the geographic distance between the place of residence of the infectors and her/his infectees) during the first wave: about 1.5 km (2.2 km if infections linked to household and school transmission are excluded). The best-fitting model entails a change in human activity regarding contacts not related to household and school. By using this model we estimate that the transmission distance sharply increased to 5.3 km (10 km when excluding infections linked to household and school transmission) during the second wave. Our study reveals a possible explanation for the observed pattern and highlights the need of better understanding human mobility and activity patterns under the pressure posed by a pandemic threat.

## Introduction

In March 2009 a novel H1N1pdm influenza virus emerged in Mexico and started spreading globally^1^. The first cases in Europe, mainly travelers coming back from infected areas (Mexico and United States), were recorded at the end of April 2009^2^. At the beginning of June 2009, more than 70 countries had been reached by the infection and the World Health Organization declared a pandemic. Because of the great attention that was paid to the pandemic threat, the surveillance of ILI cases was enhanced during the pandemic^3^ and several seroprevalence surveys were performed at different time points^4–8^. This made it possible to apply several mathematical models for epidemic spread to the available data, in order to make real-time predictions^9–11^ or to assess the most relevant factors for the observed patterns of spread^12,13^. The focus of the investigations was either on the national level or on specific epidemic hotspots^14^, and little has been ascertained about patterns of within-country epidemic spread (see however Gog *et al*.^15^ for the U.S. or Eggo *et al*.^16^ for the 1918 pandemic).

The spatial diffusion of the 2009 H1N1 influenza outbreak showed significant geographic heterogeneities^17,18^. In Europe, the pandemic progressed from West to East^12,19^, and about 66% of cases affecting European countries up to the end of June 2009 were reported in the United Kingdom^20^. Unlike all other European countries, which were characterized by moderate transmission in spring and summer and by a single fall/winter wave, in 2009, the UK experienced two waves, the first one in late spring/summer and the second one in the fall. The intensive air connections between the UK and the US and the late closure of schools for summer holidays have been identified as the main determinants of the first wave^12,21^. Data collected since 2009, together with modeling studies, have provided a clear picture of H1N1 epidemiology at the national and international scale. However, some notable patterns of spread at the sub-country level warrant further investigation. In particular, during the first wave of the pandemic, H1N1 cases were heterogeneously dispersed within England. The regions of London and West Midlands experienced early large school-based outbreaks^22,23^, and a more rapid increase of general practitioner (GP) consultation rates for influenza-like illness (ILI) with respect to the other regions^2^ - this evidence was later confirmed by a cross-sectional serological study^4^. On the contrary, the second wave spread in a much more homogeneous way, affecting most regions of England soon after the reopening of schools after the summer break^5^.

Here we aim to investigate the mechanisms that drove the observed complex pattern of spread of the 2009 influenza pandemic in England - highly localized during the first wave, rather homogeneous during the second one. In particular, the spatiotemporal spread of the pandemic could have been driven by several factors such as an increase of the transmissibility of the virus over the course of the epidemic, an increased number (or location) of the influenza cases arriving from abroad or variation in the behavior of the population. To test these (and other) hypotheses, we used previously published serological data at the regional level^4,5^ to perform a Bayesian analysis of dynamic models of influenza transmission.

## Results

We analyze seroprevalence data for England at the regional spatial resolution^4,5^ (i.e. NUTS1) by using 5 different spatially explicit individual-based models of influenza transmission calibrated through a Bayesian MCMC approach. All developed models account for influenza natural history and the sociodemographic characteristics of the population. They are structurally similar to those used for the analysis of influenza spread, containment and mitigation^12,24–28^. Briefly, all models are based on a synthetic population of agents matching socio-demographic data on age, population density, household and school structure specific for England. The transmission of the infection follows a discrete-time SLIR (Susceptible - Latent - Infectious - Removed) model, with latent period - assumed equal to the incubation period - lasting on average 1.5 days^29^ and infectious period of 1.6 days, in such a way to obtain a generation time of 3.1 days^12,30^. All models explicitly account for influenza transmission in households and schools (the latter interrupted during holidays), and transmission in all ‘other settings’ is assumed to depend on the geographic distance between individuals. In line with previous work on the 2009 H1N1 influenza, age-specific susceptibility to infection is considered as well^1,12,31,32^ - individuals aged 15+ years have a different susceptibility to infection with respect to children. The models also consider age-specific pre-pandemic immunity rates according to serosurvey data^4^. The models, however, differ from one another in a way specifically designed to test different factors that might have shaped the spread of the 2009 H1N1 influenza pandemic. The mathematical formulation of the five models is briefly described in Sec. Methods and detailed in the Supplementary Information.

### Model selection and transmission distance

We identified four factors that might have determined the observed complex pattern of spread and we tested them with four different models (M2-M5) together with a classic model (M1) based on the work by Ferguson and colleagues^25^. Specifically,

- **M1** This model is based on the classic model introduced by Ferguson and colleagues^25^. Basically M1 differs from model by Ferguson and colleagues^25^ by three factors: M1 does not explicitly consider transmission in workplaces, it does not differentiate between symptomatic and asymptomatic infectious individuals, and it explicitly considers an age-specific susceptibility to infection. Model M1 has five parameters related to virus transmissibility and susceptibility to infection: three transmission rates (in households, schools and ‘other settings’), the relative susceptibility to infection of adults with respect to children, and one multiplying factor for the transmission in ‘other settings’ while schools are closed. These parameters are epidemic-specific and thus need to be calibrated on the basis of the observed data. M1 can be considered as a reference scenario for comparing the other tested models.
  Note that the model has a distance dependent component of the force of infection in ‘other settings’ that is driven by a kernel distance function $K(d)=1/\mathrm{[1}+{(d/a)}^{\alpha }]$, where *d* is the geographic distance, *a* = 4 km and *α* = 3 as determined by Ferguson and colleagues^25^ by analyzing commuting data for the UK. Basically, the kernel defines the distance at which an infectious individual generates secondary infections through contacts in ‘other settings’ (i.e., all contacts except those occurring in households and schools).
- **M2** This model is structured exactly like model M1, except for the kernel function; here we estimate the parameter *α* regulating *K*(*d*) (and set *a* = 1 km), hypothesizing that the mean distance of infections linked to the transmission in ‘other settings’ could have been different than what was estimated by Ferguson and colleagues^25^ from commuting data, possibly because of a spontaneous human response of the population to the pandemic threat (as previously documented for the 2003 SARS epidemic^33^), leading to a very high spatial localization of the first pandemic wave.
- **M3** This model is like M2, but we let the virus transmissibility differ between the two waves by adding one free parameter regulating such a difference, either for climatic reasons or because of viral evolution, as shown by Dorigatti *et al*.^13^ for the ‘third wave’. Possibly an increase in infection transmissibility might have led to a quicker spread in the second wave and thus to a rather homogeneous seroprevalence by region.
- **M4** This model is like M2, but we added one parameter regulating the daily number of imported cases that might have been larger during the second wave. Indeed, influenza incidence was much higher in the fall than in the spring both in the United States^15^ and in the other European countries^34^, the origins of most travelers to the United Kingdom. It is then plausible that the influx of infectives was higher during the second wave, when individual case reporting had already been discontinued^3^, and this in turn might have led to a more spatially homogeneous infection spread.
- **M5** In this model, the parameter *α* regulating the infection kernel *K*(*d*) in ‘other settings’ is allowed to differ between the first and the second wave. This might have occurred because the population behaved differently at the beginning of the pandemic from later on, and this could have led to different patterns of epidemic spread. For the rest, the model is identical to M2.

A key outcome derived from the calibrated models is the transmission distance. This is defined as the distance between the place of residence of an infector and the place of residence of the infectees. The transmission distance is not a model parameter; it is instead derived from the simulations of the calibrated models. Specifically, we consider all the transmission events simulated by a specific model and, for each of them, we compute the geographic distance between the place of residence of the infector and the place of residence of the infectees. The transmission distance in ‘other settings’ is computed in the same way, but it accounts only for infection events linked to a contact occurring in settings other than households or schools. The estimated transmission distances for the five models are reported in Table 1.

**Table 1:** Estimated DIC scores and transmission distances.

Model name	Brief description of the model and hypotheses	Number of free parameters	DIC	Mean transmission distance in ‘other settings’ (and SD)^†^	Mean transmission distance (and SD)^†^
M1	Classic model based on pre-pandemic knowledge	5	434.8	w1: 11.4 km (20.3) w2: 10.8 km (19.9)	w1: 6.5 km (16.3) w2: 5.7 km (15.5)
M2	Transmission distance might be different from what was inferred from commuting data	6	426.1	w1: 2.2 km (1.7) w2: 2.2 km (1.6)	w1: 1.5 km (4.1) w2: 1.9 km (5.9)
M3	Transmission distance might be different from what was inferred from commuting data; virus transmissibility might be different in the two waves	7	454.0	w1: 2.1 km (0.9) w2: 2.1 km (0.8)	w1: 1.4 km (5.4) w2: 1.9 km (6.8)
M4	Transmission distance might be different from what was inferred from commuting data; import of cases might be larger in the second wave	7	438.5	w1: 2.1 km (0.6) w2: 2.1 km (0.7)	w1: 1.5 km (2.9) w2: 1.8 km (5.9)
M5	Transmission distance might be different from what was inferred from commuting data and might be different in the two waves	7	390.9	w1: 2.1 km (0.7) w2: 10.0 km (29.1)	w1: 1.4 km (3.8) w2: 5.3 km (20.7)

^†^Values refer to the first (w1) and second (w2) pandemic waves.

In this respect, Table 1 shows that there is a consensus between all the analyzed models with regards to the estimated transmission distance in ‘other settings’ during the first wave of the pandemic. In fact, all models (except for M1, where the kernel regulating the distance is kept fixed) estimate a mean transmission distance in ‘other settings’ around 2 km, which decreases to about 1.5 km if all sources of infections are considered. This figure is remarkably different from what we found by using the “classic” model (M1), which estimates a transmission distance in ‘other settings’ slightly above 11 km in agreement with the commuting distance traveled by UK residents (i.e., 15 km^35^). The probability distributions of the transmission distance for models M1-M5 are reported in the Supplementary Information. It is worth noting that all models provide similar estimates for the five (common) parameters regulating virus transmissibility and susceptibility to infection (see Supplementary Information).

Deviance Information Criterion (DIC)^36^ is used to compare the five models. According to this criterion, the model obtaining the best score is M5 (see Table 1). This supports the hypothesis that the transmission distance in ‘other settings’ changed over the course of the pandemic.

**Figure 1:**
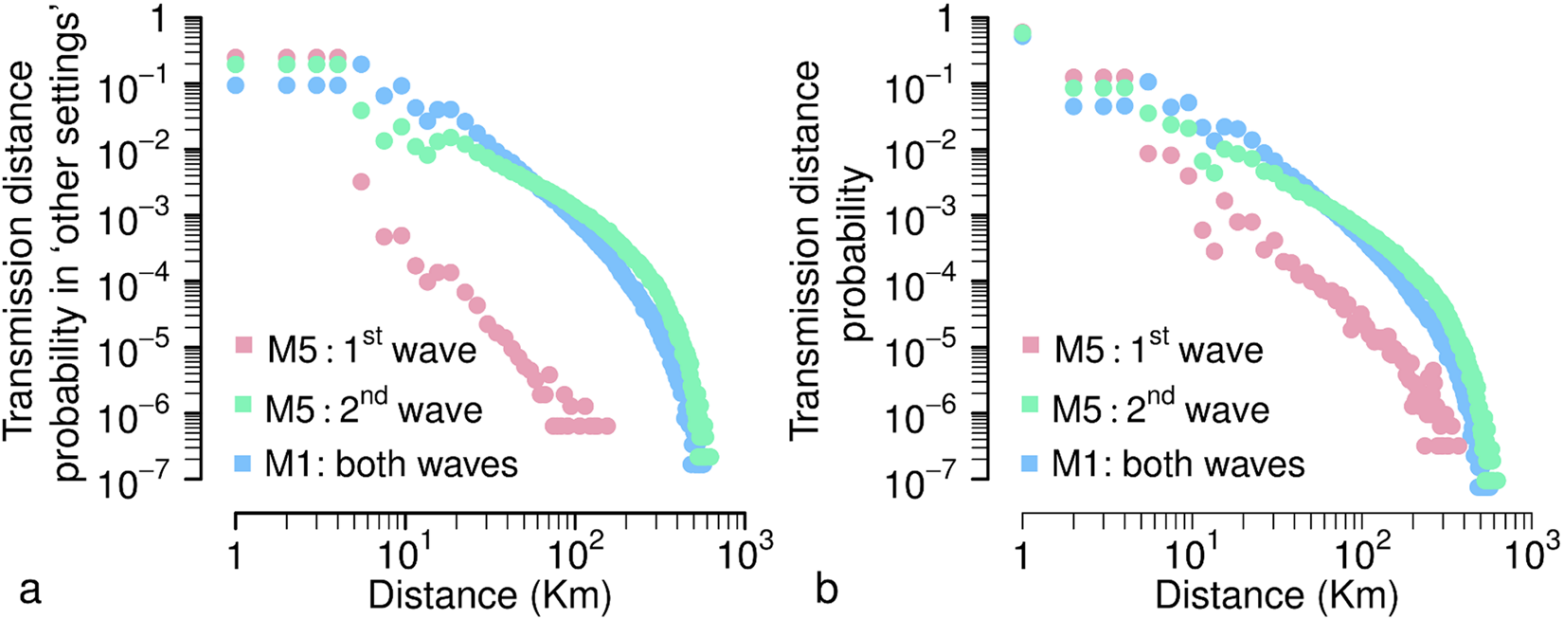
Transmission distance. (**a**) Probability distribution of transmission distance in ‘other settings’ as estimated by model M5 for the two influenza waves and by model M1 for both waves combined together (results separated by wave for M1 are reported in Supplementary Information). Note that the transmission distance depends on both kernel function and population density (which is responsible for the bumps in the curves). The curves were obtained by averaging over 100 simulations run by using median values of the posterior distributions of model parameters; distances have been grouped in 1 km intervals and displayed at the superior limit of the intervals, so that for instance 1 on the *x*-axis corresponds to secondary infections occurring between 0 km (e.g., infection between members of the same household) and 1 km. (**b**) Probability distribution of transmission distance (i.e., accounting for infections occurring in all settings).

Intuitively, Model M5 estimates a high force of infection due to close distance contacts (roughly inside a radius of 5 km) and a lower force of infection at higher distances during the first wave, and a less sharp decline of force of infection with distance in the fall wave (Fig. 1a). In particular, the mean transmission distance in ‘other settings’ was estimated to have increased from 2.1 km (SD = 0.7) during the early epidemic phase to 10.0 km (SD = 29.1) later on. The latter is close to the mean commuting distance reported to the UK Department of Transport^35^, namely 15 km, and aligned with what was estimated by model M1 that is based on the kernel estimated by Ferguson and colleagues^25^ before the 2009 pandemic (i.e., 10.8 km, see Table 1, model M1). If we consider the mean transmission distance irrespective of the setting where the infection occurs, estimates provided by model M5 become: 1.4 km (SD = 3.8) during the first wave, 5.3 km (SD = 20.7) during the second wave (Fig. 1b).

In summary, our results highlight that i) transmission occurred at a markedly lower distance during the initial phase of the pandemic, and ii) during the second wave the estimated transmission distance is close to the value observed in the commuting flows.

From now on, we will primarily focus on model M5 as it best explains the observed data.

### Geographic spread of the pandemic

**Figure 2:**
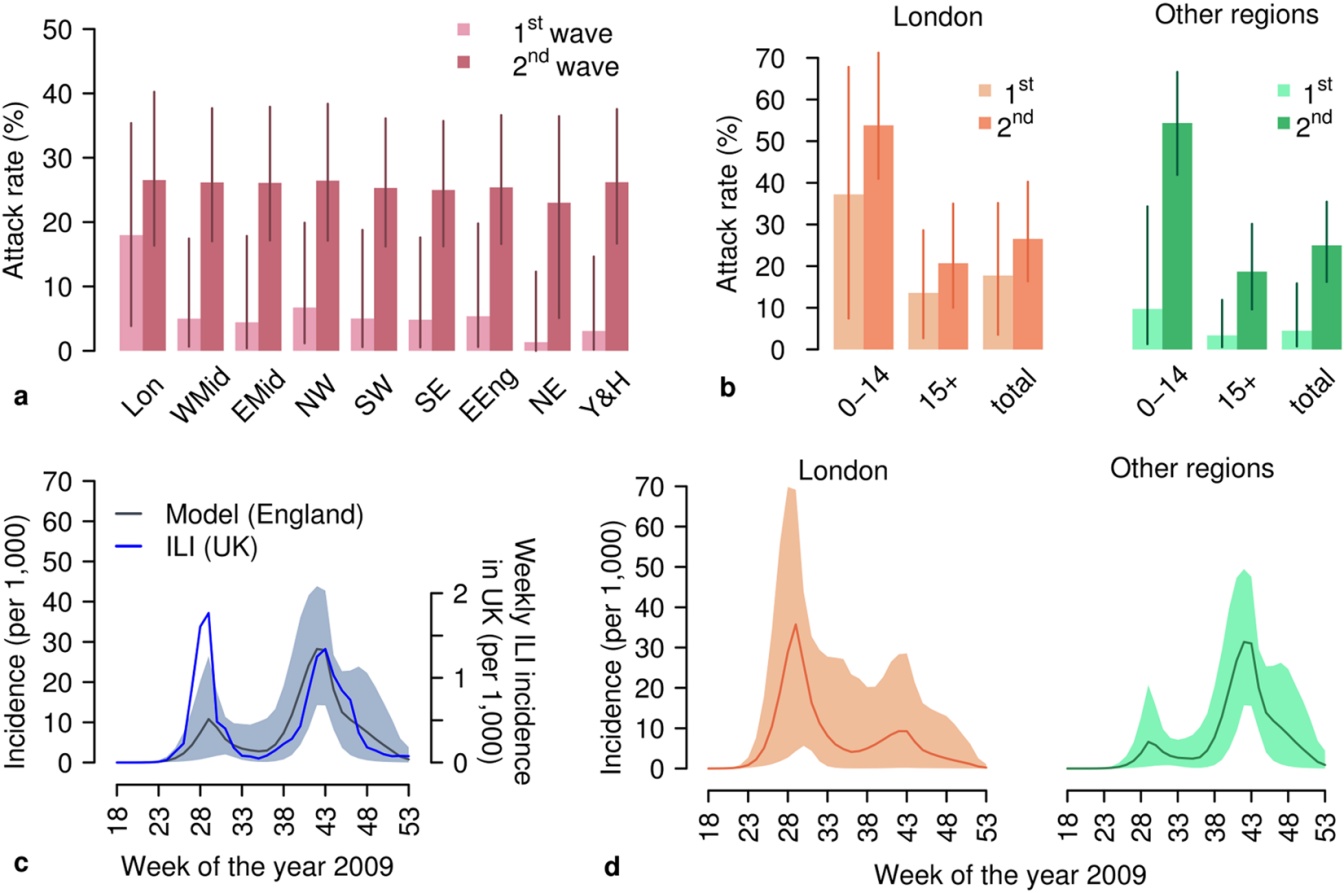
Spatiotemporal dynamics of 2009 H1N1 influenza pandemic in England. (**a**) Estimated attack rate (mean and 95%CI) by region of England at the end of the two epidemic waves as resulting from model M5. Abbreviations used are: Lon, London; WMid, West Midlands; EMid, East Midlands; NW, North West; NE, North East; SW, South West; SE, South East; EEng, East of England; Y&H, Yorkshire & Humber. (**b**) Estimated attack rate by age (mean and 95%CI) in London and in all other England regions at the end of the first wave and at the end of the second wave as resulting from model M5. (**c**) Weekly incidence of new reported ILI cases in the UK^12^ and weekly incidence of new infections estimated by simulating the calibrated model M5 (mean and 95%CI) for England. Note that the comparison can be considered representative for the timing of the epidemic only, and not for the absolute magnitude of incidence, as: i) ILI cases refer to the entire UK, and ii) they are affected by underreporting (which was estimated to be remarkably lower during the first epidemic wave^13,38^, than in the second one). (**d**) Weekly incidence of new infections in London and in all other regions of England (grouped together) as estimated by model M5 (mean and 95%CI).

By simulating the calibrated model M5, we found a substantial variability in incidence rates during the first wave between the regions of England (Fig. 2a). Such a heterogeneous pattern is confirmed by both the analysis performed by the Strategic Health Authorities^37^ and serological data^4,5^. On the contrary, at the end of the pandemic, estimated and actual prevalence rates^5^ show a rather homogeneous pattern across all of England (see Fig. 2a). The same pattern can also be found by looking at age-specific prevalence rates (Fig. 2b).

At the national level, the pandemic clearly showed two waves of infection (Fig. 2c). According to our modeling analysis and in agreement with previous investigations^13^, the second wave was characterized by a markedly higher peak weekly incidence (mean 28.2, 95%CI: 14.2–43.9 cases per 1,000 individuals, to be compared with 10.8, 95%CI: 1.2–26.4 cases per 1,000 individuals for the first wave - Fig. 2c). The crude number of notified ILI cases shows the opposite pattern; however, this has been explained due to a higher (three to ten times) GP consultation rate during the first wave^13,38^.

**Figure 3:**
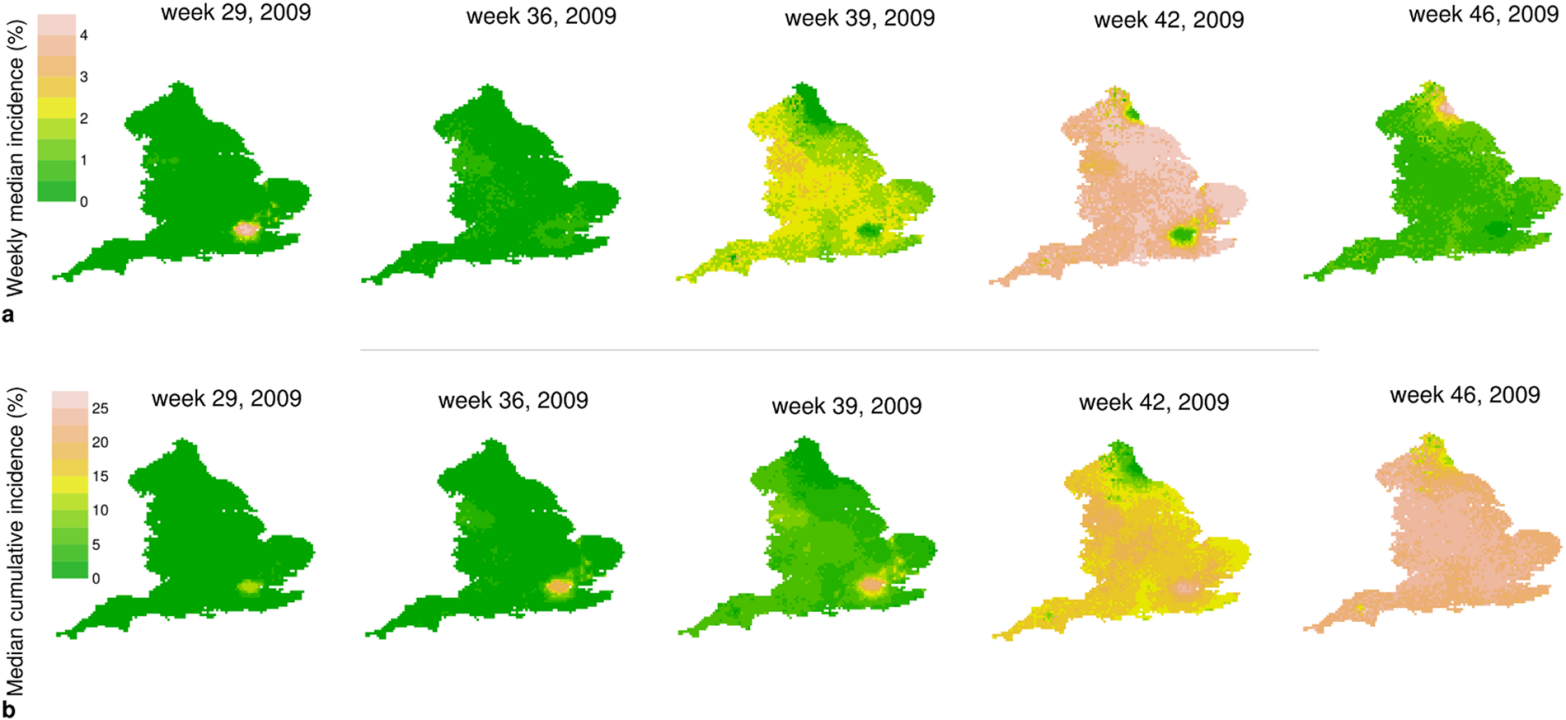
Simulated geographic spread of 2009 H1N1 influenza pandemic in England. (**a**) Simulated weekly incidence of new infections in each single cell (median over 2,000 simulations) as resulting from model M5. (**b**) Simulated cumulative weekly incidence of new infections in each single cell (median over 2,000 simulations) as resulting from model M5. The maps were generated using Grass GIS 6.4.2 (https://grass.osgeo.org/announces/announce_grass642.html).

By looking more closely at the sub-national scale, the observed dynamics at the national scale are determined, according to our modeling analysis, by the sum of two very different dynamics at the regional level: London suffered a major epidemic wave during the spring/summer and a more moderate one during the fall; the opposite pattern can be observed in the other regions of England (Fig. 2d). Such a pattern is also clearly visible by looking at the dynamics of weekly and cumulative incidence at the resolution of single geographic cell (Fig. 3a,b and the Supplementary Video S1). Moreover, as shown in the Supplementary Video S1, it is apparent that the fall wave spread through the country in a remarkably more homogeneous way and at a higher pace than the summer one. These patterns are driven by the detected change in the transmission distance: a lower distance during the early phase corresponds to a lower rate of spatial diffusion of the infection, while the estimated larger value in subsequent phases corresponds to a quicker and much more homogeneous geographic spread.

Support to the findings obtained from model M5 come from the other analyzed models. We found a consensus between all five models with respect to several aspects of the geographic spread of the pandemic. In particular, all models estimate two major influenza waves at the national scale. At the sub-national scale, the first wave is more localized in the area of London (although to a different extent depending on the considered model), while at the end of the pandemic the estimated final attack rate is rather homogenous in all regions. The differences between the models mainly lay in the factors driving the pattern of spread. For instance model M4 estimate an unrealistically large number of imported cases to explain the pattern, while model M3 requires a remarkable influenza activity during the summer, which appears not well supported by epidemiological evidence. Results for models M1-M4 are discussed in more detail in the Supplementary Information.

### Epidemiological characterization of the pandemic

**Figure 4:**
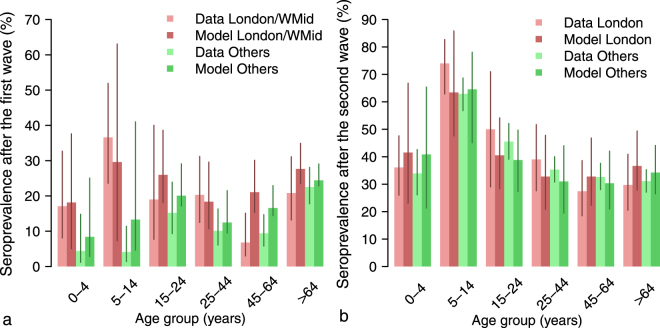
Epidemiological characteristics of the 2009 H1N1 influenza pandemic in England. (**a**) Age-specific seroprevalence (mean, 95% CI) by age group and region as reported in^45^, (proportion of serum samples with haemagglutination inhibition titre 1:32 or more) and as estimated by model M5, as of August 2009 (i.e., at the end of the first epidemic wave). Regions are grouped as in the original works^4,5^; WMid corresponds to West Midlands. (**b**) as a, but as of January 2010 (i.e., at the end of the second wave).

According to seroprevalence data^4,5^, a substantial proportion of adults and elderly were already protected against H1N1 infection and most of the cases in 2009 occurred among school-age children; the same pattern is estimated by model M5 (Fig. 4a,b). Model simulations also suggest that individuals older than 15 years were less susceptible to infection than younger ones: the estimated age-specific susceptibility to infection of adults is 0.61 (95%CI: 0.28–0.94), compared with a baseline value of 1 for children. Such an estimate is in line with previous findings where adults were found to be approximately half as susceptible as younger individuals^1,8,31,38^.

A second major determinant of epidemic spread is the reproduction number (i.e., the average number of secondary cases generated by an index case). We estimated a relatively low transmissibility of the H1N1pdm virus compared to previous pandemics: effective reproduction number of 1.45 (95% CI: 1.36–1.54) for the first wave and 1.30 (95% CI: 1.08–1.52) early on in the second wave. Both estimates are in line with results reported in previous independent studies and reviewed by Biggerstaff and colleagues^39^.

The limited transmissibility of the H1N1pdm virus is not sufficient to justify the low overall attack rate measured during the first wave; rather, it is a consequence of the drop of transmission associated with the closure of schools for the summer holidays, even though we found an increase in the transmission in ‘other settings’ (representing all contacts except those occurring in household and school - see Sec. Material and Methods), possibly ascribable to increased activity of students outside schools. In particular, we estimated an increase of 1.2 (95% CI: 0.6–1.9), which is well aligned with findings obtained from the analysis of seasonal influenza in France^40^.

Although schools remained closed during summer (as well as in fall and winter breaks), they had a major role in the spread of infection. In particular, considering the whole pandemic, we estimated that 17.8% (95% CI: 2.7–35.6) of the infections are linked to contacts at school - this is remarkable considering that the fraction of school-age individuals in England corresponds to only 20% of the population. Moreover, we estimated that 34.7% (95% CI: 4.7–54.1) of infections occurred in households, and 47.5% (95% CI: 19.7–79.2) in ‘other settings’. Comparable values have been obtained in a previous work on the 2009 H1N1 pandemic in Italy^41^, and for past influenza pandemics as well^25^.

Models M1-M4 provide similar estimates of epidemiological indicators with respect to those resulting from the analysis of model M5. Briefly, all models estimate a lower susceptibility to infection of adults with respect to children, ranging, on average, from 0.49 to 0.61. There is a strong consensus between the models in the value of the reproduction number of the first wave (averages ranged from 1.42 of model M1 to 1.45 of model M5), while in the second wave the estimates are slightly more variable (averages ranged from 1.22 of model M1 to 1.38 of model M3, which however assumes an increased transmissibility of the virus over the course the pandemic). Although highly variable, also the fractions of infection by setting are rather consistent between the models. The estimated parameters and epidemiological indicators for the five models are reported in Tables S4 and S5 in the Supplementary Information.

## Discussion

In this work we conducted a Bayesian analysis of the spatiotemporal dynamics of the 2009 H1N1 influenza pandemic in England at a sub-national scale. Specifically, we tested different hypotheses that could explain the observed highly localized spread during the first wave of the pandemic and the subsequent homogeneous diffusion during the second one. The mean transmission distance in settings other than households and schools has been estimated in all the analyzed models to be, during the first pandemic wave, remarkably lower (≈2 km) than the mean commuting distance (≈15 km). On the other hand, the model best reproducing the data highlights that such a distance increased substantially over the course of the pandemic (the estimated mean distance for the second pandemic wave was 10 km). In addition, our findings provide a clear picture of the epidemiology of the 2009 pandemic, in agreement with the existing knowledge. Children younger than 15 years were the most affected age group: other studies found that children have higher contact rates^42,43^ and a lack of pre-existing immunity^4,5,8,44^; here we also estimated a higher (about twice as much) susceptibility to infection compared with adults, similar to previous works on the 2009 H1N1 pandemic^1,8,31^. We estimated a relatively low value of the reproduction number (around 1.4) in both waves. We also provided quantitative estimates of the proportion of cases by setting that is crucial for determining the effectiveness of intervention options, such as school closure^28,45,46^, highlighting a major contribution of schools to the overall transmission. Consistent with serum sample data^5^, we estimated that London experienced a marked first wave, while other regions showed little evidence of a summer wave, and that the fall wave was lower in London than elsewhere.

What is notably novel in this study is the detection of a clear signal that the transmission distance was lower than previously thought and perhaps also changed over time. In particular, during the initial phase of the epidemic, when the attention of the public on the pandemic threat was higher, we found that infections were generated at a much closer distance than the average commuting distance. In the following phases, we estimate that the radius of diffusion of the epidemic became comparable with the distance traveled by commuters. A possible explanation for such a pattern lies in behavioral changes, spontaneously emerging as a response to the pandemic threat. During an emerging epidemic people may engage in precautionary behaviors that alter the transmission dynamics of the disease^47,48^. An initial overestimation of the risk of infection has been detected in Italy^49^ and Mexico^50^. Moreover, according to survey results^51,52^, in the case of an influenza pandemic a large proportion of people are willing to avoid crowded places, especially public transportation. For instance, a worker who usually commutes to reach his workplace, would likely continue to travel the same distance to reach the workplace even during a pandemic, but he might decide to avoid crowded environments near the workplace (such as pubs, and restaurants) where he usually goes outside business hours. Our findings suggest that in the early phase of the pandemic behavioral changes may have led to either a reduction of mobility in absolute terms or, more likely, to a decrease in the number of potentially infectious contacts at high distance from the place of residence/study.

Our results support the idea already presented by Birrell *et al*.^53^ that the geographic spread of influenza might be inaccurately described by raw commuting fluxes. However, although our results point in the direction of a human behavioral adaptation to the pandemic threat, they are far from conclusive. In fact, other factors could have been responsible for the changes in the force of infection that we measured. Clearly, data quality (far from being flawless) and (un)availability impose several limitations on our modeling analysis. First, concerning the initial seeding of the infection, we compute the region-specific probability of importing cases by using data on the total volume of incoming passengers by airport, disregarding their origin and final destination. Second, our model neglects cross-border infections that, given the geographic features of England, may have been imported from Scotland or through the Channel Tunnel, i.e. from France and continental Europe. A further limitation of the model is that transmission in workplaces is not explicitly modeled; in fact, its contribution is included in the ‘other settings’ component of the force of infection. However, the contribution of workplaces during the 2009 pandemic has been shown to be marginal^41^. Finally, it should be remarked that describing infection transmission through a distance kernel neglects most details of human mobility, although it has been shown that it gives an adequate description, especially in a setting like that of the UK^54^.

In conclusion, our results help to shed light on the epidemiology of the 2009 H1N1 pandemic and provide a possible explanation for the initially heterogeneous spatial spread of the epidemic within England, followed by a highly homogeneous one. Our analysis calls for a deeper understanding of human interactions and movements under the pressure posed by an epidemic threat. This would be instrumental for the design of more effective control strategies and revising current preparedness plans, particularly in light of the recent surge in the number of human H7N9 cases and deaths^55^.

## Methods

### The models

All the models used in this work are individual-based, spatially explicit, stochastic models of influenza transmission in England, adapted from previous models developed for Europe^12,26^. The five models can be seen as nested models of a more general formulation. Full details on model structure and calibration are available in the Supplementary Information.

Remarkably, we explicitly model transmission in schools and households, i.e., the two most important settings for transmission according to the literature on the 2009 influenza pandemic^12,41^. Transmission in ‘other settings’, which accounts for contacts occurring in the general community (e.g., workplaces, means of transport, free-time activities, etc.) is shaped by the decreasing kernel function of the distance $K(d)=1/\mathrm{[1}+{(d/a)}^{\alpha }]$, where *d* is the geographic distance, and *a* and *α* regulate the kernel. In particular,

- for model M1, we used *a* = 4 km and *α* = 3 as determined by Ferguson and colleagues^25^ by analyzing commuting data for the UK;
- for models M2-M4, we assumed *a* = 1 km and estimate *α*;
- for model M5, we used *α* = (*α*_1_, *α*_2_), where *α*_1_ regulates the kernel in the first pandemic wave (here defined as the period since the start of the epidemic up to week 33, 2009) and *α*_2_ in the second one (from week 34, 2009 until the end of the epidemic), and assumed *a* = 1 km (as for models M2-M4).

The kernel function is based on the idea that the distance traveled follows a power law, as suggested by several studies on human mobility (see for instance Brockmann *et al*.^56^ and Song *et al*.^57^). We assumed a fixed value for *a* in models M2-M5 in order to decrease the number of free parameters to be estimated; the reason why parameter *a* is not set to the same value in all models is discussed in the Supplementary Information.

Each student is assigned to a specific school, which is determined on the basis a resource competition model^58^. The resulting mean distance from home to school is 4.0 km, in agreement with the observed data according to the Department of Transport^35^, i.e. 4.3 km.

All models explicitly includes the closure of schools during holidays, according to the 2009 school calendar for England. During these periods transmission in schools is interrupted, whereas transmission in ‘other settings’ is assumed to increase, as in^12,40^. Simulations start on April 27, 2009, the day of the first cases reported in England. Infection is seeded within the population according to the actual time series of reported travel-related cases^59^. Once a case is imported, its location in a specific region is proportional to the volume of incoming air passengers per region over the period April-June 2009 as provided by the Civil Aviation Authority^60^; within each region, imported cases are distributed proportionally to population density.

### Model calibration

Model calibration was performed by using Markov chain Monte Carlo (MCMC) sampling applied to the binomial likelihood of the age-specific and region-specific prevalence of H1N1 antibodies observed in England according to serum samples collected in August 2009^4^ (i.e., at the end of the first wave) and over the period January-April 2010^5^ (i.e., after the end of the second wave). All models share a set of five common parameters, i.e., three transmission rates (in households, schools and ‘other settings’), the relative susceptibility to infection of adults with respect to children, and one multiplying factor for the transmission in ‘other settings’ while schools are closed. M3 has one further free parameter determining the (possible) increase of virus transmissibility during the second wave. M4 has one parameter regulating the number of imported cases during the second wave. Models M2-M4 have one free parameter regulating the kernel function *K*(*d*) (i.e., *α*). Finally, model M5 has one parameter (*α*_1_) regulating kernel *K*(*d*) during the first wave and a second one (*α*_2_) for the second wave. Details on model calibration are provided in the Supplementary Information.

### Data availability statement

Data used to develop and validate the model can be retrieved through the referenced works. Outputs of model simulations are available upon request.

## Electronic supplementary material

Supplementary Information

Supplementary Video S1
