# Supplementary material for: Detecting a Surprisingly Low Transmission Distance in the Early Phase of the 2009 Influenza Pandemic
Source: Sci Rep. 2017 Sep 26;7:12324. doi: 10.1038/s41598-017-12415-2 (PMC5615056; doi:10.1038/s41598-017-12415-2)
Supplement: Supplementary file 1 — Supplementary Information [file 41598_2017_12415_MOESM1_ESM.pdf]

Detecting a Surprisingly Low Transmission Distance in the Early Phase of the 2009 Influenza Pandemic

Valentina Marziano[1,2], Andrea Pugliese[2], Stefano Merler[1] and Marco Ajelli[3,1]

[1] Bruno Kessler Foundation, Trento, Italy,
[2] Department of Mathematics, University of Trento, Trento, Italy,
[3] Laboratory for the Modeling of Biological and Socio-technical Systems, Northeastern University, Boston, MA, USA

Supplementary Information

Contents

1 Materials and methods **2**
1.1 Socio-demographic model 2
1.2 Infection transmission model 2
1.3 Seeding of infection 6
1.4 Description of the five models and their assumptions 7
1.5 Model calibration 8
1.6 Computation of R_{e} 10

2 Additional results **11**
2.1 Estimated models parameters and epidemiological indicators 11
2.2 Estimated transmission distance 12
2.3 Geographic spread of the pandemic estimated by models M1-M4 16

3 Supplementary Video S1 **21**

1 Materials and methods

The models used in this work are stochastic spatially-explicit individual-based models of influenza transmission for England adapted from previous models developed for Europe [1, 2, 3]. Each model is the combination of two different layers: i) a model of the socio-demographic structure, and ii) an infection transmission model. All models share the same socio-demographic model, while differ in some details of the infection transmission process as detailed in the next sections. (Note that all the five models are nested in a more general model, as detailed in Sec. 1.2).

1.1 Socio-demographic model

The socio-demographic structure of the model is the same as in [4] and we refer to this work for all technical details.

Briefly, socio-demographic data on the age structure of the population of England are used to generate about 47 millions individuals, which are distributed on a grid of 3894 cells proportionally to the population density derived from the Gridded Population of the World, version 3 (GPW v3). Each individual is characterized by an age and belongs to a household. Students are also assigned to a specific school (see below). Each household and school has specific geographic coordinates within the cell.

Household members are determined using a heuristic model previously proposed in [4], which reproduces data on household size, composition and age of household members by size specific for the United Kingdom, as provided by the Statistical Office of the European Commission (`http://ec.europa.eu/eurostat`), preserving realistic age difference between members of the same household. As shown in [4], age distribution of the population, household size, household composition by size and age distribution of household members by size simulated by using this model match the observed ones.

Schools are allocated proportionally to population density and their size is determined using data on school size by level specific for England [5]. School age children (5-18 years) are assigned to a school of the corresponding education level using the resource competition algorithm introduced in [6], which accounts for the population density of the considered geographic area. The mean home-to-school distance obtained by using this algorithm results 4 km, in excellent agreement with the value reported to the Department of Transport, namely 4.3 km [7].

1.2 Infection transmission model

The transmission of influenza infection follows a discrete-time individual-based SLIR (susceptible - latent - infectious - removed) model with time step $\Delta t = 1$ day. The model explicitly considers transmission in households, schools, and 'other settings' (which accounts for contacts occurring in all settings other than households and schools – e.g., contacts in the general community, workplaces, means of transport, free-time activities).

Simulations are initialized by including age-specific pre-pandemic immunity, as measured in [8]. In particular, for each individual i aged a_i, we determine whether he/she is initially immune by sampling from a Bernoulli distribution of probability given by the age-specific pre-pandemic immunity reported in [8].

At any time step of the simulation each susceptible individual i has a probability $p_i(t) = 1-e^{-\Delta t \lambda_i(t)}$ of becoming infected. The probability of infection, which is re-computed at each time step, depends on the individual risk of infection $\lambda_i(t)$ that takes into account the contribution of infection sources in each of the considered settings:

- contacts with infectious members of his/her household;
- contacts with infectious schoolmates, if individual i is a student;
- contacts with infectious people in 'other settings'.

Similarly to [2, 9], transmission in 'other settings' explicitly depends on the geographic distance, which is modeled by a kernel distance function K (the definition of K is given below).

The generalized formulation of the risk of infection (and thus also of the transmission model) is defined by the following equation:

$$\begin{aligned}\lambda_i(t) \quad = \quad & \rho(a_i)\left[\sum_{\{k=1,\dots,N|H_k=H_i\}} \frac{\tilde{\chi}(t)\beta_h I_k}{(n_i-1)^\omega}\right. \\ & + \sum_{\{k=1,\dots,N|S_k=S_i\}} \mu(t)\frac{\tilde{\chi}(t)\beta_s I_k}{m_i} \\ & \left. + \sum_{\{k=1,\dots,N\}} \tilde{\tau}(t)\frac{\tilde{\chi}(t)\beta_r I_k K(d_{ik})}{\sum_{\{j=1,\dots,N\}} K(d_{jk})}\right]\end{aligned}$$

where

- N is the population of England.
- H_i is the index of the household of individual i, n_i is the number of members of the household and $\omega = 1.63$, as estimated in [10], in order to take into account of contact heterogeneity in the household network.
- S_i is the index of the school where individual i studies and m_i is the school size, if individual i is a student.
- $I_k = 1$ if individual k is infected, 0 otherwise.
- a_i is the age of individual i.
- $\rho(a_i)$ is the age-dependent susceptibility to infection. For the sake of simplicity, we divide the population into two groups: children ($a_i < 15$ years), for which $\rho(a_i) = 1$, and adults ($a_i \geq 15$ years), for which $\rho(a_i) = \sigma \in [0, 1]$. In the manuscript we refer to σ as the relative susceptibility to infection of adults with respect to children.
- β_h is the transmission rate within households (day^{-1}).
- β_s is the transmission rate within schools (day^{-1}).

- β_r is the transmission rate in 'other settings' (day^{-1}).
- $K(d_{ij})$ is a decreasing function of the geographic distance d_{ij} between the households of individuals i and j, modeling human mobility patterns. In particular, we assume that function K has the following form:

$$K(d_{ij}) = \begin{cases} \frac{1}{1+\left(\frac{d_{ij}}{a}\right)^{\alpha_1}} & \text{up to week 33, 2009 (i.e., during the first pandemic wave)} \\ \frac{1}{1+\left(\frac{d_{ij}}{a}\right)^{\alpha_2}} & \text{from week 34, 2009 (i.e., during the second pandemic wave)} \end{cases} \quad (1)$$

  i.e. function K is shaped by the parameters a, α_1 and α_2. Intuitively, parameter a mainly regulates the shape of the function at short distances, whereas parameters α_1 and α_2, mainly shape the exponential decay at medium/long distances in the two considered time windows.

- $\mu(t)$ accounts for the school calendar, not allowing transmission at school when schools are closed. In particular,

$$\mu(t) = \begin{cases} 0 \text{ if } t \in \{\text{July 20, 2009 - September 1, 2009 (summer break)}\} \\ 0 \text{ if } t \in \{\text{October 26, 2009 - October 31, 2009 (fall break)}\} \\ 0 \text{ if } t \in \{\text{December 19 - January 3, 2010 (winter break)}\} \\ 1 \text{ otherwise} \end{cases}$$

- $\tilde{\tau}(t)$ shapes influenza transmissibility in 'other settings' during school closures, accounting for the fact that students might be more active in 'other settings' during those periods [11]. In particular,

$$\tilde{\tau}(t) = \begin{cases} \tau \in [0, +\infty) \text{ if } t \in \{\text{July 20, 2009 - September 1, 2009 (summer break)}\} \\ \tau \in [0, +\infty) \text{ if } t \in \{\text{October 26, 2009 - October 31, 2009 (fall break)}\} \\ \tau \in [0, +\infty) \text{ if } t \in \{\text{December 19 - January 3, 2010 (winter break)}\} \\ 1 \text{ otherwise} \end{cases}$$

- $\tilde{\chi}(t)$ regulates possible changes in virus transmissibility between the two waves. In particular,

$$\tilde{\chi}(t) = \begin{cases} \chi = 1 \text{ if } t < \text{week 33, 2009 (first wave)} \\ \chi \in [0, +\infty) \text{ if } t \geq \text{week 34, 2009 (second wave)} \end{cases}$$

At any time step Δt of the simulation latent individuals become infectious at a rate $\Delta t/T_L$, where T_L is the latent period - assumed equal to the incubation period - that lasts on average 1.5 days [12]. Infectious individuals recover (and are assumed to be fully protected) at a rate $\Delta t/T_I$, where T_I is the infectious period, which lasts on average 1.6 days in such a way to obtain a generation time of 3.1 days, in agreement with the scientific literature on the 2009 H1N1 pandemic [2, 13]. The distributions of the latent and infectious period obtained by assuming a time-step of 1 day

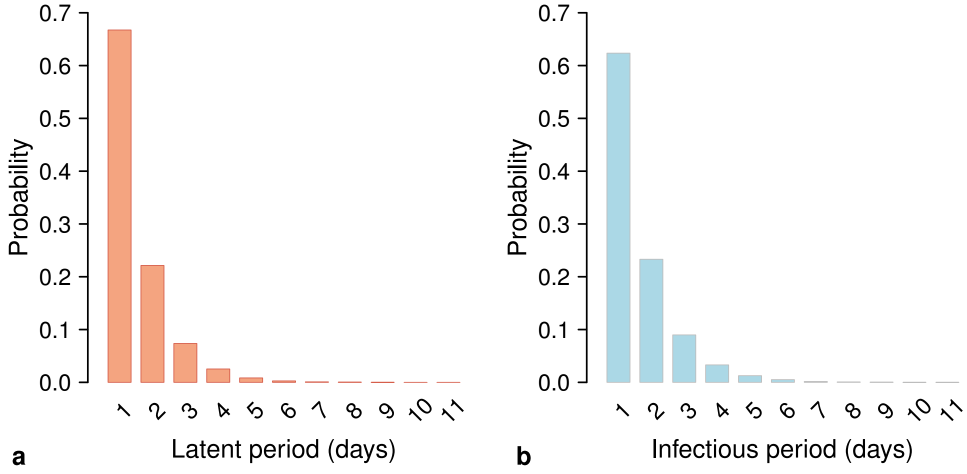

Figure S1: **Latent and infectious period distributions. a** Probability distribution of the length of the latent period as obtained by using a time-step of 1 day. **b** Probability distribution of the length of the infectious period as obtained by using a time-step of 1 day.

are shown in Figure S1. Note that the actual distributions of latent and infectious periods are presumably far from exponential: for instance, Cori et al. [14] using viral excretion data from volunteers challenged with different influenza strains, estimated for the latent period a mean of 1.63 days with a standard deviation of 0.26, and for the infectious period a mean of 0.99 days with a standard deviation of 0.96. In this sense, the standard deviation of the generation time arising from simulating the model with a time-step of 1 day may be closer to the actual one than the standard deviation obtained under the assumption of exponential distributions (see Table S1). Moreover, using a shorter time-step would pose serious computational challenges.

Table S1: Mean and standard deviation (SD) of exponentially distributed length of the latent and infectious period distributions for different values of the time-step.

Time-step	Latent period		Infectious period	
	Mean (days)	SD (days)	Mean (days)	SD (days)
Continuous	1.5	1.5	1.6	1.6
0.25 day	1.5	1.4	1.6	1.5
0.5 day	1.5	1.2	1.6	1.3
1 day	1.5	0.9	1.6	1.0

1.3 Seeding of infection

The daily number of imported cases in the first wave is based on the actual time-series of travel-related cases reported in [16]. Previous studies on the 2009 H1N1 pandemic influenza have highlighted that H1N1 importations in the early phase of the 2009 pandemic were strongly correlated with air passenger flows [17, 18]. We thus determine the geographic location of imported cases by randomly distributing cases among all English regions (i.e. NUTS1) proportionally to air passenger arrivals. In particular, we use data on incoming air passenger flows during the period April-June 2009 provided by the Civil Aviation Authority [15] to determine the fraction of incoming passengers specific for each region of England. As shown in Figure S2, the fraction of incoming passengers is not proportional to population density, with Greater London accounting for about 70% of the total volume. For each time step, the number of imported cases is distributed among regions proportionally to the corresponding fraction of incoming passengers and, within each region, cases are assigned to a cell proportionally to population density.

The daily number of cases imported during the second wave is sampled at each time-step from a Poisson distribution with parameter $\lambda = 10^{\delta}$-1, where $\delta \in [0, +\infty]$. The geographic location of cases imported in the second wave is determined by using the same procedure described for the first wave.

We tested an alternative method for determining the destination of imported cases. Specifically, we assume to randomly distribute imported cases proportionally to the population density of each cell (Figure S2**b**). Specifically, we calibrated a new model, which is exactly as model M2 (see main text and Sec. 1.4 of this document), but differing for two details: i) the model is calibrated on serological seroprevalence data on the first pandemic wave only [8], and ii) we assume importation of cases proportional to population density. Our modeling results show that

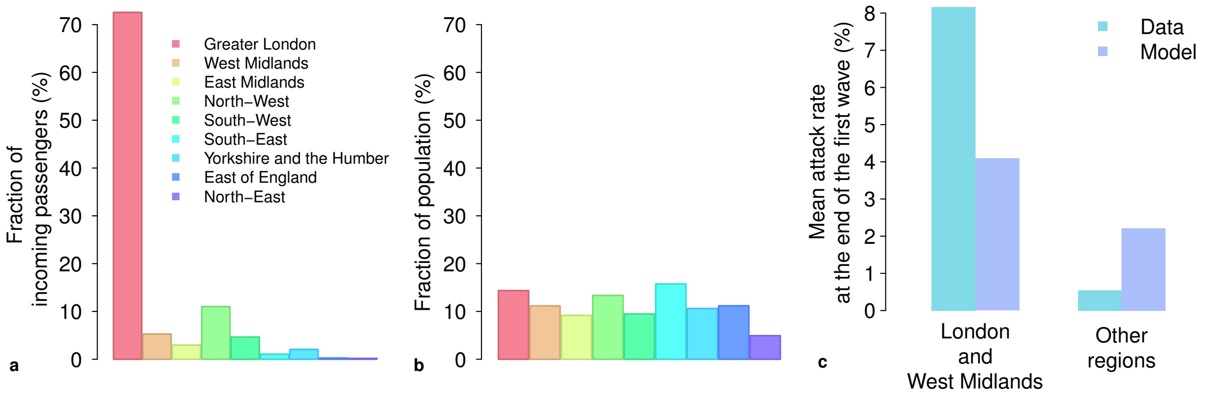

Figure S2: **Incoming air passengers and population density. a** Fraction of incoming air passengers by region (%) based on data provided by the Civil Aviation Authority [15] on incoming air flows by airport. **b** Fraction of population by region (i.e. NUTS1) of England as obtained from Eurostat. **c** Mean attack rates at the end of the first wave as resulting from seroprevalence data [8] (light blue) and as estimated by the model (blue). The model used to produce this result is as model M2 (see main text and Sec. 1.4 of this document), but it is calibrated on the data on first pandemic wave only and assumes importation of cases proportional to the population (i.e., as in panel b).

the hypothesis of import of cases proportional to population density data leads to a regional distribution of cases during the first wave not compliant with the observed data [8] (see Fig. S2c). As the model was not able to properly reproduce data on the first pandemic wave, we decided not to consider this model for further analyses.

1.4 Description of the five models and their assumptions

The five models considered in this work test different hypotheses on the factors that might have shaped the complex pattern of spread observed during the 2009 influenza pandemic in England. The models are obtained from the general formulation as follows:

M1 The parameter values regulating the kernel function (see Equation 1) are fixed at $a = 4$ km and $\alpha_1 = \alpha_2 = 3$, as estimated by Ferguson and colleagues [9] from the analysis of commuting data for the UK. $\chi = 1$ for both pandemic waves and $\delta = 0$, i.e. no changes in transmissibility of the virus are considered, nor the importation of additional cases during the second wave is considered. Note that in this model the transmission distance is determined by human mobility patterns as estimated from commuting data for the UK.

M2 Parameter a regulating the kernel function (K) is fixed at $a = 1$ km, whereas $\alpha_1 = \alpha_2 =: \alpha$ is estimated, $\chi = 1$ for both pandemic waves and $\delta = 0$. This model tests the hypothesis that the observed pattern of spread might be explained by an alteration in the transmission distance with respect to that derived from commuting data.

M3 Parameter a is fixed at $a = 1$ km, whereas $\alpha_1 = \alpha_2 =: \alpha$ is estimated, $\chi = 1$ for the first wave, whereas it is estimated for the second wave, and $\delta = 0$. This model tests the hypothesis that the observed pattern of spread might be explained by an alteration in the transmission distance during the pandemic and by an increase in the transmissibility of the virus during the second wave.

M4 Parameter a is fixed at $a = 1$ km, whereas $\alpha_1 = \alpha_2 =: \alpha$ is estimated, $\chi = 1$ for both waves and δ is estimated. This model tests the hypothesis that the observed pattern of spread might be explained by an alteration in the transmission distance during the pandemic and by an increasing number of imported cases during the second wave.

M5 Parameter a is fixed at $a = 1$ km, whereas α_1 and α_2 are estimated separately, where α_1 regulates the kernel function in the first wave and α_2 in the second wave; $\chi = 1$ for both pandemic waves and $\delta = 0$. This model tests the hypothesis that the observed pattern of spread might be explained by an alteration in the transmission distance during the first wave and by a second change occurring during the second wave (possibly coming back to a value close to that obtained by the analysis of commuting data).

Note that model M1 assumes $a = 4$ km, while models M2-M5 assume $a = 1$ km. Model M1 assumes $a = 4$ km in order to be consistent with the work of Ferguson and colleagues [9] that we decided to use as reference model. However, fixing $a = 4$ km in models M2-M5 would impose a constraint on the fraction of infections within a certain distance, which is an undesired feature for our analysis aimed at estimating the transmission distance directly from seroepidemiological

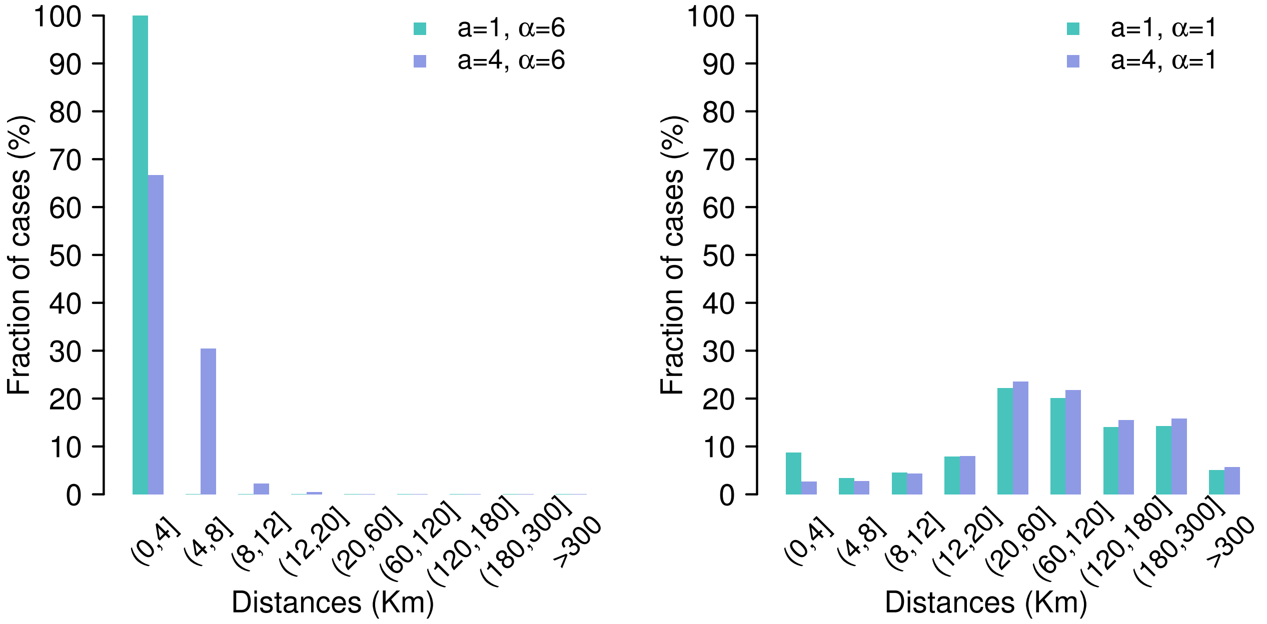

Figure S3: **Transmission distance a** Fraction of secondary infections (%) generated at a certain distance as obtained by fixing $\alpha = 6$ and setting $a = 1$ km (green) and $a = 4$ km (purple). **b** As **a**, but fixing $\alpha = 1$ and setting $a = 1$ km (green) and $a = 4$ km (purple).

data. Fig.S3a shows that, by setting $a = 4$ km, even by using $\alpha = 6$, a value much larger than those estimated for human mobility [9, 19, 20], a considerable fraction of secondary infections occur at a distance larger than 4 km from the place of residence of the infector, while this is not the case when $a = 1$ km. On the other hand, assuming $a = 1$ km does not impose any constraint on the maximum transmission distance, provided that appropriate values of α are used (Fig.S3b).

1.5 Model calibration

Model calibration is performed using Markov chain Monte Carlo (MCMC) sampling applied to the product of the binomial likelihoods ($\mathcal{L}_1$ and $\mathcal{L}_2$) of the age-specific prevalence of H1N1 antibodies observed in England respectively in August 2009 [8] (after the first wave) and in the period January-April 2010 [21] (after the second wave). Data are disaggregated into the groupings of regions specified in Table S2.

The likelihood function $\mathcal{L}_1$ is defined as

$$\begin{aligned} \mathcal{L}_1(n_m^{\mathrm{A}}, r_m^{\mathrm{A}}, n_m^{\mathrm{B}}, r_m^{\mathrm{B}}|\Theta) = \prod_{m \in M} & \frac{n_m^{\mathrm{A}}!}{r_m^{\mathrm{A}}!(n_m^{\mathrm{A}} - r_m^{\mathrm{A}})!} (p_m^{\mathrm{A}}(\Theta))^{r_m^{\mathrm{A}}} (1 - p_m^{\mathrm{A}}(\Theta))^{n_m^{\mathrm{A}} - r_m^{\mathrm{A}}} \\ & \frac{n_m^{\mathrm{B}}!}{r_m^{\mathrm{B}}!(n_m^{\mathrm{B}} - r_m^{\mathrm{B}})!} (p_m^{\mathrm{B}}(\Theta))^{r_m^{\mathrm{B}}} (1 - p_m^{\mathrm{B}}(\Theta))^{n_m^{\mathrm{B}} - r_m^{\mathrm{B}}} \end{aligned} \quad (2)$$

where

- Θ is the vector of free parameters;
- M is the set of age groups considered in [8];
- n_m^{A} is the number of individuals in the m-th age group in Group A in the post-first wave dataset [8];
- r_m^{A} is the number of seropositive individuals (haemagglutination inhibition titre 1:32 or more) in the m-th age group observed in Group A in the post-first wave dataset [8];
- $p_m^{\mathrm{A}}(\Theta)$ is the seroprevalence in the m-th age group simulated by the model with parameter set Θ in Group A after the first wave;
- n_m^{B} is the number of individuals in the m-th age group in Group B in the post-first wave dataset [8];
- r_m^{B} is the number of seropositive individuals (haemagglutination inhibition titre 1:32 or more) in the m-th age group observed in Group B in the post-first wave dataset [8];
- $p_m^{\mathrm{B}}(\Theta)$ is the seroprevalence in the m-th age group simulated by the model with parameter set Θ in Group B after the first wave.

$\mathcal{L}_2$ is defined analogously to $\mathcal{L}_1$, but using the post-second wave dataset [21] and the respective grouping of regions (see Table S2).

All models share a set of five common parameters, i.e. three transmission rates (in households, β_h, schools, β_s, and 'other settings', β_r), the relative susceptibility to infection of adults (individuals aged ≥ 15 years) with respect to children (σ) and the multiplying factor for the transmission in 'other settings' while schools are closed (τ). Model M3 has one further free parameter determining the (possible) increase of virus transmissibility during the second wave (χ). Model M4 has one parameter regulating the daily number of imported cases during the second wave (δ). Models M2-M4 have one free parameter regulating the kernel distance function $K(d)$, i.e. α. Finally, model M5 has one free parameter (α_1) regulating the kernel function $K(d)$ for

Table S2: Grouping of regions in the datasets used for model calibration.

**Calibration dataset**	**Group A**	**Group B**
Post-first wave [8]	London	East Midlands
	West Midlands	North-West
		South-East
		South-West
Post-second wave [21]	London	East Midlands
		North-West
		South-East
		South-West
		West Midlands

the first wave and a second parameter (α_2) for the second wave. The vectors of free parameters Θ corresponding to each model are reported in Table S3.

Table S3: Set of free parameters for the different models considered.

**Model**	Θ
M1	$(\beta_h, \beta_s, \beta_r, \sigma, \tau)$
M2	$(\beta_h, \beta_s, \beta_r, \sigma, \tau, \alpha)$
M3	$(\beta_h, \beta_s, \beta_r, \sigma, \tau, \alpha, \chi)$
M4	$(\beta_h, \beta_s, \beta_r, \sigma, \tau, \alpha, \delta)$
M5	$(\beta_h, \beta_s, \beta_r, \sigma, \tau, \alpha_1, \alpha_2)$

The posterior distribution of Θ is determined using random-walk Metropolis-Hastings sampling [22]. For each of the five models we run four chains, each one having a different starting point. Each chain is based on 22,000 realizations. To perform the simulations of the calibrated models, for each model we considered only one chain (i.e., one starting point), we discarded the first 2,000 iterations (considered as burn-in period), and we run 20,000 realizations each one having the parameter set estimated by MCMC. We assume non-informative prior distributions on all model parameters (i.e., the prior distribution for β_s is a flat distribution in $[0, 1000]$). On the contrary, for the transmission rate in households we assume a prior uniform distribution in $[0, 3]$ according to the findings reported in [10]. Indeed, when assuming $\beta_h = 3$, the household secondary attack rate is more than twice the one estimated for the 2009 influenza pandemic [10], thus providing an upper bound for the household transmission rate.

1.6 Computation of R_e

The effective reproduction number R_e represents the average number of infections generated by an infectious individual in a partly immune population. We estimated its value for the first and second wave from the corresponding epidemic growth rate [2, 9, 23, 24].

Briefly, the exponential growth rates r_1 and r_2 of the two epidemic waves are estimated by fitting a linear model to the logarithm of influenza incidence over the the week of maximum growth. r_1 is computed on the basis of simulated data up to week 33, 2009, and r_2 on the basis of simulated data after week 34, 2009. The effective reproduction number is thus computed as:

$$R_e^i = (1 + r_i T_L)(1 + r_i T_I) \qquad i \in \{1, 2\}$$

where $T_L = 1.5$ days and $T_I = 1.6$ days are the average duration of the latent and infectious period, respectively. The details on the derivation of such an equation can be found for instance in [24].

2 Additional results

2.1 Estimated models parameters and epidemiological indicators

Estimated values of all parameters for the five analyzed models are reported in Tab. S4. The key epidemiological quantities obtained by each model, i.e. the effective reproduction numbers for the first and second wave, and the fraction of cases by setting after the first and the second wave, are summarized in Tab. S5.

Symbol	Description	Model M1 (mean and 95%CI)	Model M2 (mean and 95%CI)	Model M3 (mean and 95%CI)	Model M4 (mean and 95%CI)	Model M5 (mean and 95%CI)
β_h	Transmission rate in households (days^{-1})	1.72 (95% CI: 0.17-2.92)	1.35 (95% CI: 0.07-2.9)	1.65 (95% CI: 0.15-2.92)	1.53 (95% CI: 0.1-2.94)	1.75 (95% CI: 0.11-2.93)
β_s	Transmission rate in schools (days^{-1})	0.62 (95% CI: 0.11-1.18)	0.66 (95% CI: 0.11-1.23)	0.76 (95% CI: 0.16-1.38)	0.65 (95% CI: 0.08-1.36)	0.72 (95% CI: 0.16-1.22)
β_r	Transmission rate in "other settings" (days^{-1})	0.56 (95% CI: 0.18-1.46)	0.7 (95% CI: 0.24-1.88)	0.46 (95% CI: 0.1-1.36)	0.72 (95% CI: 0.2-1.69)	0.57 (95% CI: 0.18-1.41)
Σ	Susceptibility to infection of adults (≥15 years) as compared to children (<15 years)	0.59 (95% CI: 0.24-0.95)	0.58 (95% CI: 0.22-0.95)	0.59 (95% CI: 0.23-0.96)	0.49 (95% CI: 0.2-0.93)	0.61 (95% CI: 0.28-0.94)
τ	Scale factor for transmission in "other settings" during school holidays	1.34 (95% CI: 0.64-1.96)	1.05 (95% CI: 0.56-1.9)	1.33 (95% CI: 0.58-1.95)	1.27 (95% CI: 0.57-1.96)	1.21 (95% CI: 0.61-1.94)
α_1	Kernel parameter (in the 1st wave for model M5)	-	3.56 (95% CI: 0.08-5.89)	3.92 (95% CI: 0.42-5.87)	4.03 (95% CI: 0.6-5.9)	4.06 (95% CI: 0.53-5.86)
α_2	Kernel parameter 2nd wave	-	-	-	-	2.18 (95% CI: 0.11-5.39)
χ	Scale factor of transmission rates in the 2nd wave	-	-	1.16 (95% CI: 0.75-1.84)	-	-
δ	Parameter determining the daily number of imported cases C in the 2nd wave ($C=10^{\delta}-1$)	-	-	-	1.87 (95% CI: 0.11-4.75)	-

Table S4: **Estimated values of models parameters.**

Description	Model M1 (mean and 95%CI)	Model M2 (mean and 95%CI)	Model M3 (mean and 95%CI)	Model M4 (mean and 95%CI)	Model M5 (mean and 95%CI)
Effective reproduction number (1st wave)	1.42 (95% CI: 1.34-1.51)	1.44 (95% CI: 1.36-1.54)	1.43 (95% CI: 1.33-1.54)	1.44 (95% CI: 1.37-1.54)	1.45 (95% CI: 1.36-1.54)
Effective reproduction number (2nd wave)	1.22 (95% CI: 1.1-1.37)	1.26 (95% CI: 1.05-1.47)	1.38 (95% CI: 1.07-1.81)	1.23 (95% CI: 1.05-1.47)	1.3 (95% CI: 1.08-1.52)
Cases in households (%), 1st wave	36.6 (95% CI: 6.6 -53.9)	32.9 (95% CI: 3.2-52.5)	37.6 (95% CI: 6.6-56)	32.5 (95% CI: 2.9-53.7)	38 (95% CI: 4.7-53.9)
Cases in schools (%), 1st wave	7.4 (95% CI: 0.6-31.2)	10.4 (95% CI: 0.6-33.8)	17.9 (95% CI: 1.6-43.8)	9.2 (95% CI: 0.5-37.7)	11.9 (95% CI: 1.1-32.2)
Cases in 'other settings' (%), 1st wave	55.3 (95% CI: 25.2-86.7)	56.3 (95% CI: 26.1-89.3)	44.6 (95% CI: 11.3-80.1)	57.2 (95% CI: 22.8-90.1)	50.7 (95% CI: 22.8-84.8)
Cases in households (%), 2nd wave	37.6 (95% CI: 6.8-53.9)	32.5 (95% CI: 3.1-53.1)	38.5 (95% CI: 6.5-56.0)	32.7 (95% CI: 3.1-53.1)	38.4 (95% CI: 4.7-54.1)
Cases in schools (%), 2nd wave	13.8 (95% CI: 1.8-34.4)	14.5 (95% CI: 1.6-35.6)	21.6 (95% CI: 3.6-41.5)	14.3 (95% CI: 1.2-41)	17.4 (95% CI: 2.7-35.6)
Cases in 'other settings' (%), 2nd wave	49 (95% CI: 22.6-80.8)	52.7 (95% CI: 24.4-86.4)	41.1 (95% CI: 14.0-74.3)	52.8 (95% CI: 21.2-86.0)	45.7 (95% CI: 19.7-79.2)

Table S5: **Epidemiological indicators as estimated by models M1-M5.**

2.2 Estimated transmission distance

For models M2-M5 (i.e., all models except model M1 for which the kernel parameters are kept fixed), we estimated very narrow kernels for the first wave (see Tab. S4). This resulted in a low average transmission distance (see Table 1 in the Main Text) and in a sharply decreasing distribution (see Fig. S4). However, although the narrow kernel, the models estimated a homogeneous attack rate within the entire England at the end of the pandemic. For instance, if we consider a variant of model M5, where $\alpha_1 = \alpha_2 = 4.06$ and not considering school closures during holidays (all the other model parameters are those estimated for model M5 and reported in Tab. S4), we found that the pandemic spreads in an heterogeneous way (see the different peak times for England regions reported in Fig. S5). However, at the end of the epidemic the final attack rate by region is very homogenous (see Fig. S6). Indeed, the pandemic is capable to reach all regions because of the combination of contacts in the general community and because of commuting from the place of residence to school. This is further supported by the analysis of models M2-M4. In fact, slight variations in the transmission distance are observed when all sources of infections are considered (Fig. S4b), suggesting a larger importance of school transmission later on in the epidemic (see also the fraction of cases by setting reported in Tab. S5), when the infection started to reach a larger geographical area. On the other hand, the estimated transmission distance in 'other settings' does not remarkably change over time according to models M2-M4 (Fig. S4a). Another factor contributing to the homogenous final attack rate by region is the increased transmission rate in 'other settings' during the summer school closure, which both contributed to keep influenza circulating and increased the chance of generating secondary infections outside the network of close contacts of household members and schoolmates.

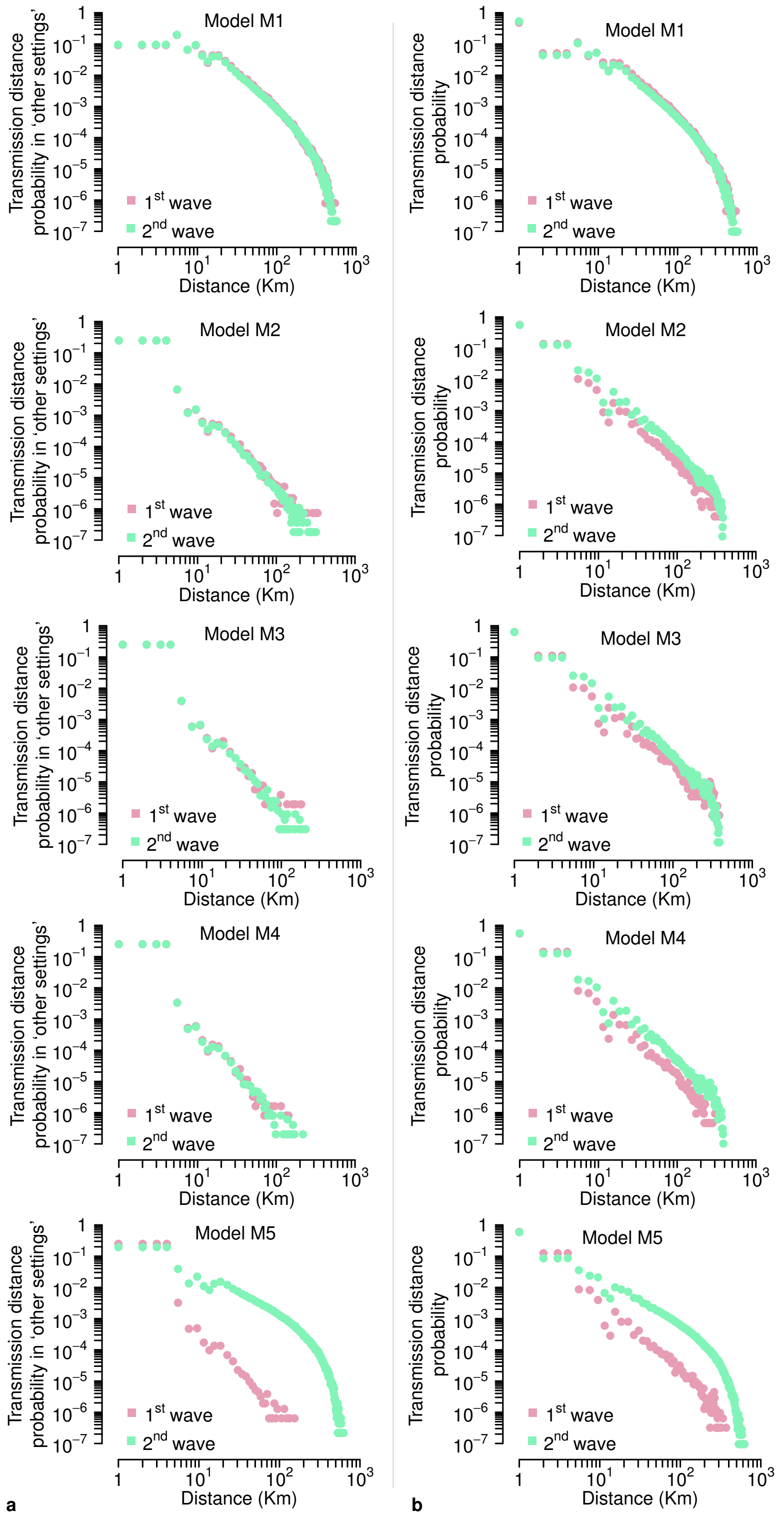

Figure S4: **Estimated transmission distance for the different models.** Probability distributions for models M1-M5 of transmission distance in 'other settings' (panel a) and overall transmission distance (panel b).

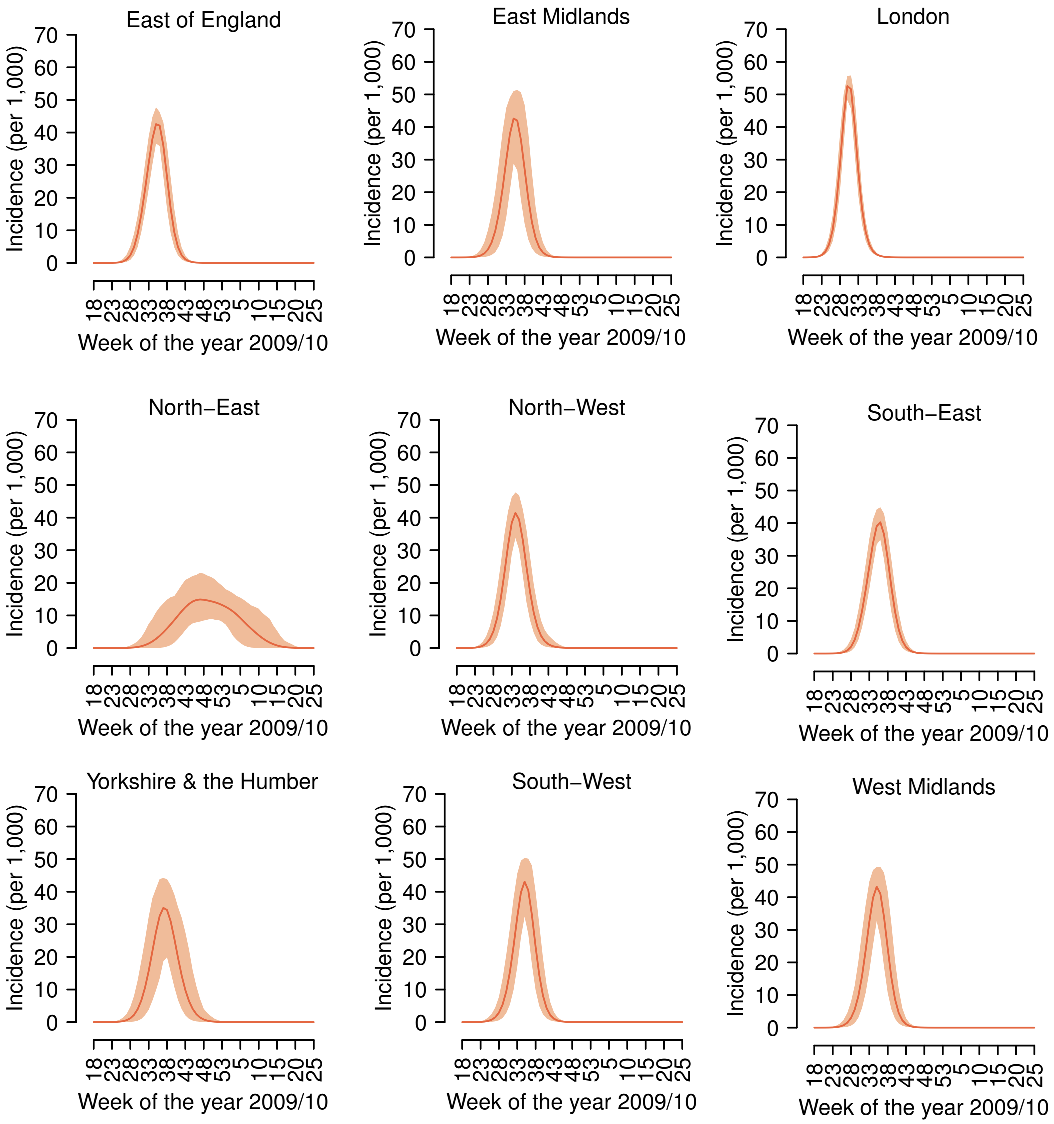

Figure S5: **Spatial spread of the 2009 H1N1 influenza pandemic in England estimated by a variant of model M5.** Weekly incidence of new infections by region (mean and 95%CI) as estimated by model M5, where $\alpha_1 = \alpha_2 = 4.06$ and without considering holidays school closures (other model parameters are those estimated for model M5 and reported in Tab. S4).

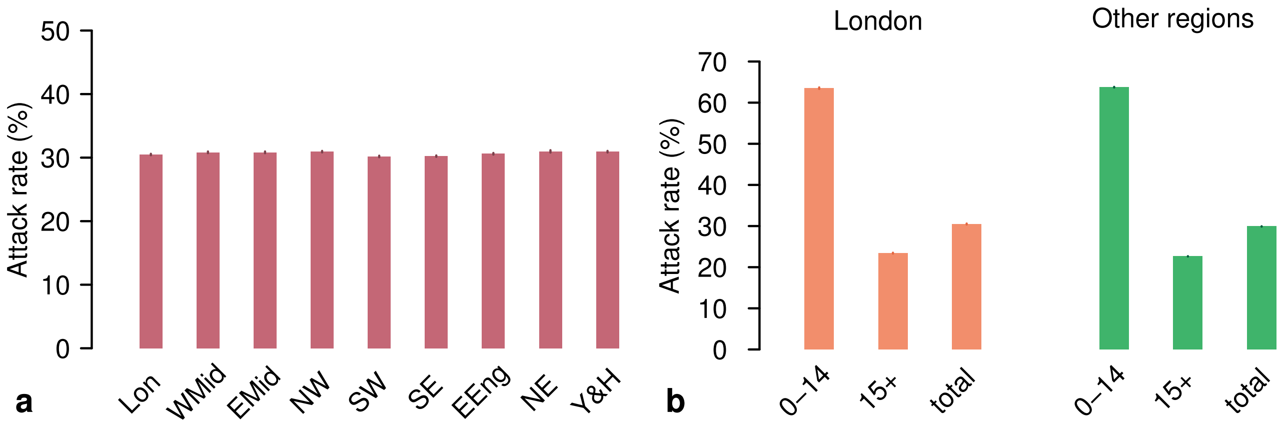

Figure S6: **Final attack rates by region estimated by a variant of model M5.** **a** Estimated attack rate (mean and 95%CI) by region of England at the end of the epidemic. Abbreviations used are: Lon, London; WMid, West Midlands; EMid, East Midlands; NW, North West; NE, North East; SW, South West; SE, South East; EEng, East of England; Y&H, Yorkshire & Humber. **b** Estimated attack rate by age (mean and 95%CI) in London and in all other England regions at the end of the pandemic. Both panels are obtained by simulating model M5, where $\alpha_1 = \alpha_2 = 4.06$ and without considering holidays school closures (other model parameters are those estimated for model M5 and reported in Tab. S4).

2.3 Geographic spread of the pandemic estimated by models M1-M4

Model M1 estimates a rather homogenous spread of the pandemic in both waves (Fig. S7). In particular only a slight difference in the prevalence between London and West Midlands, and the other regions is estimated, especially if compared with the estimates of model M5 (compare Fig. S7a with Fig. 4a in the main text). Influenza activity during the summer is estimated to be rather high, which appears to be less compliant with ILI reported by the HPA than what estimated by model M5 (compare Fig. S7c with Fig. 2c in the main text). Nonetheless, the difference in the pandemic dynamics between London and the other regions of England is still clearly visible (Fig.S7d,e).

The geographic spread of the pandemic estimated by model M2 is very close to that estimated by model M5 (compare Fig. S8 with Fig. 2 and 4 in the main text). Indeed for model M2 we estimated a very narrow kernel, similar to the one estimated for model M5 in the first wave (see Tab. S4), supporting the finding that the transmission distance was very low at least in the early phase of the pandemic. The epidemics simulated by model M2 then lead to a rather homogenous attack rate across England regions, partially thanks to a larger fraction of cases linked to contacts in 'other settings' (Tab. S4 and S5).

The geographic spread of the pandemic estimated by model M3 is reported in Fig. S9. Model M3 estimates a slightly more homogenous spread during the first wave (Fig. S9a with Fig. 4a in the main text)) than model M5 and a markedly large influenza activity during the summer, especially in London area (Fig. S9). The final attack rate estimated by model M3 is quite compliant with that estimated by all the other models.

The geographic spread of the pandemic estimated by model M4 is reported in Fig. S10. Model M4 estimates a quite heterogeneous pattern across England regions during the first wave, very similar to that estimated by model M5 (compare Fig. S10a with Fig. 4a in the main text) and an homogenous attack rate by region at the end of the pandemic (compare Fig. S10b with Fig. 4b in the main text). However, model M4 also estimate a remarkable influenza activity during the summer (Fig. S10c,d,e), and an unreasonably large number of imported influenza cases over time (see inset in Fig. S10d).

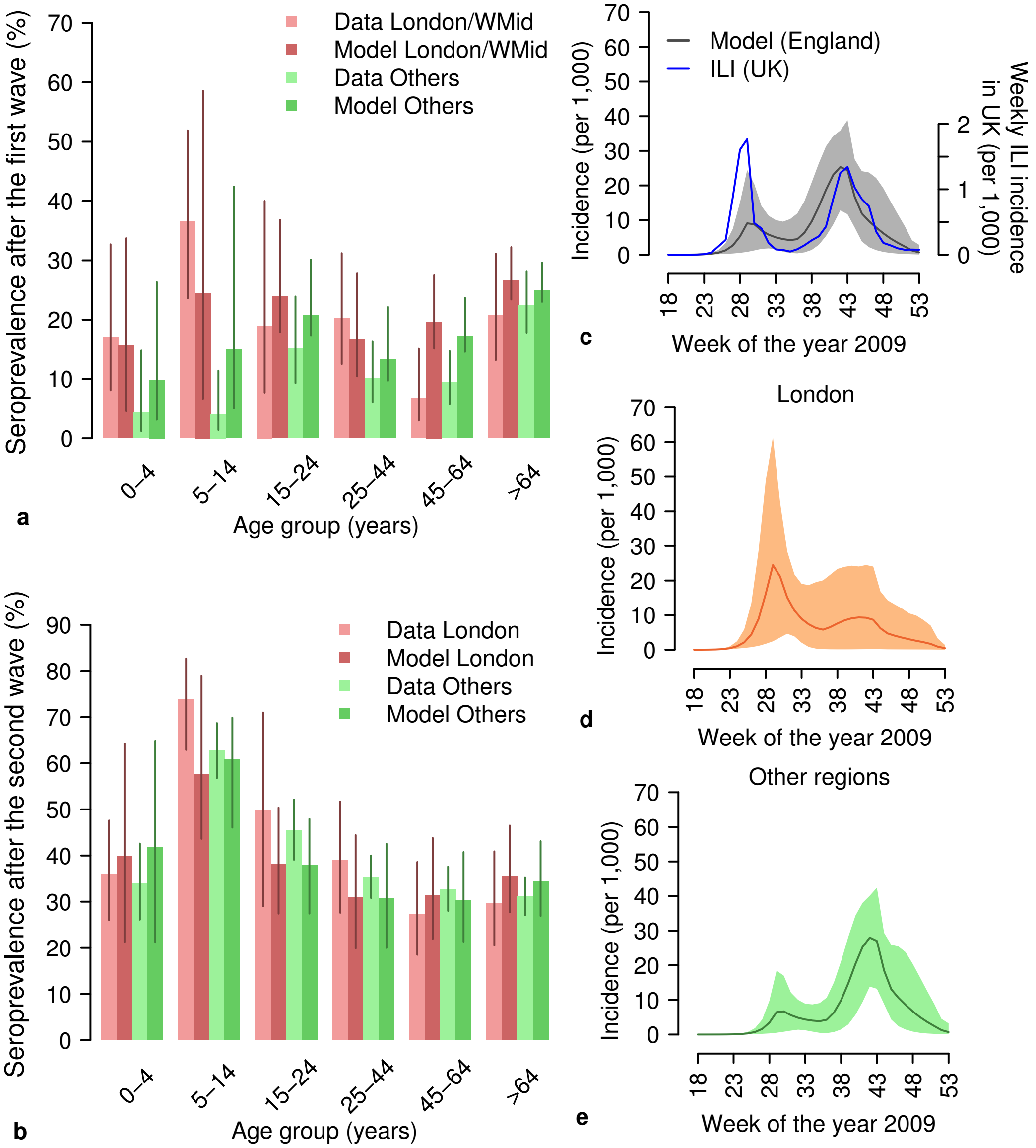

Figure S7: **Spatial spread of 2009 H1N1 influenza pandemic in England as estimated by model M1.** **a** Age-specific seroprevalence (mean, 95% CI) by age group and region as reported in [8, 21] (proportion of serum samples with haemagglutination inhibition titre 1:32 or more) and as estimated by model M1, as of August 2009 (i.e., at the end of the first epidemic wave). Regions are grouped as in the original works [8, 21]; WMid corresponds to West Midlands. **b** as **a**, but as of January 2010 (i.e., at the end of the second wave). **c** Weekly incidence of new reported ILI cases in the UK [2] and weekly incidence of new infections estimated by simulating the calibrated model M1 (mean and 95%CI) for England. **d** Weekly incidence of new infections in London as estimated by model M1 (mean and 95%CI). **e** The same as **d** but for all other regions of England (grouped together).

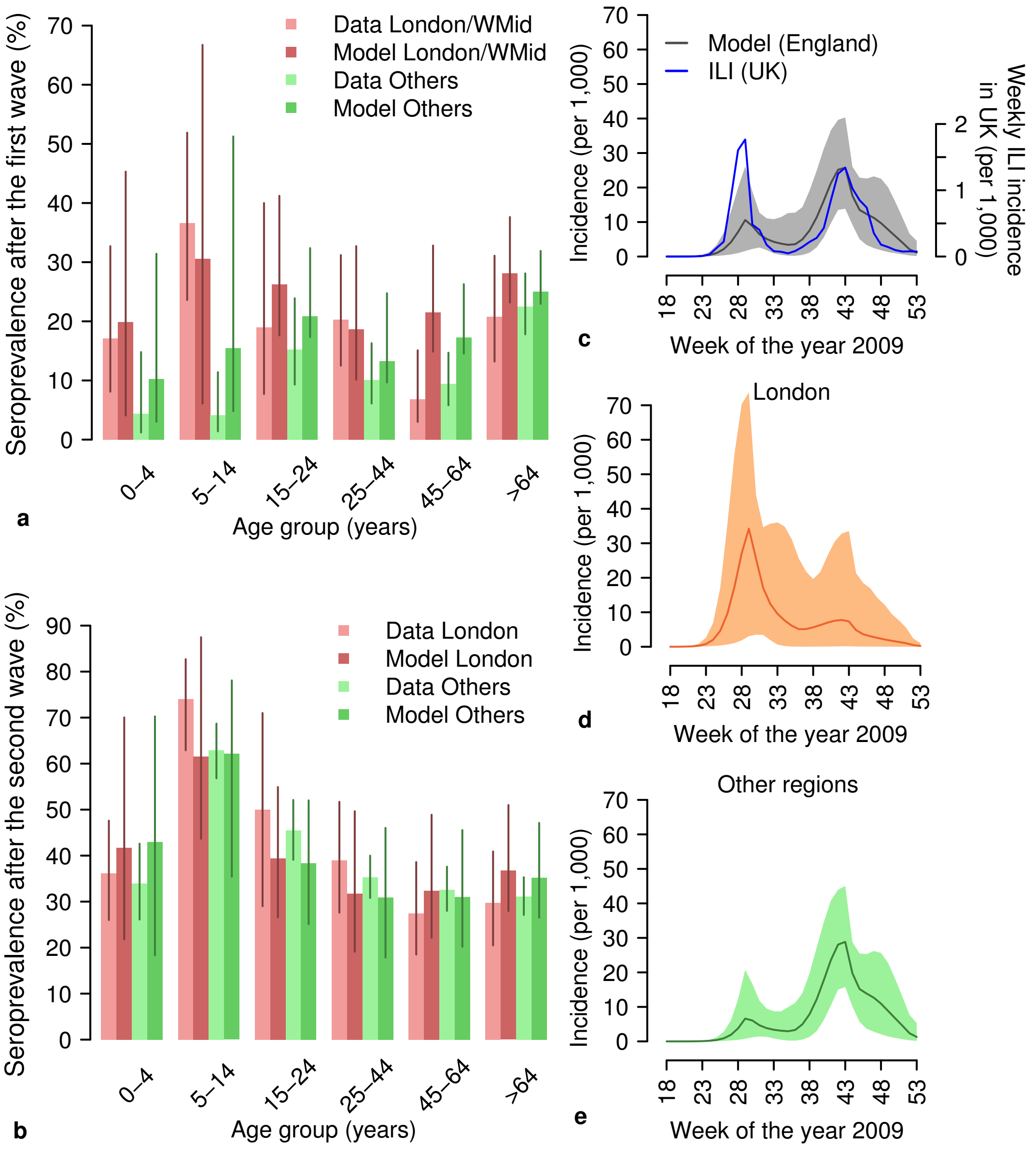

Figure S8: **Spatial spread of 2009 H1N1 influenza pandemic in England as estimated by model M2. a** Age-specific seroprevalence (mean, 95% CI) by age group and region as reported in [8, 21] (proportion of serum samples with haemagglutination inhibition titre 1:32 or more) and as estimated by model M2, as of August 2009 (i.e., at the end of the first epidemic wave). Regions are grouped as in the original works [8, 21]; WMid corresponds to West Midlands. **b** as **a**, but as of January 2010 (i.e., at the end of the second wave). **c** Weekly incidence of new reported ILI cases in the UK [2] and weekly incidence of new infections estimated by simulating the calibrated model M2 (mean and 95%CI) for England. **d** Weekly incidence of new infections in London as estimated by model M2 (mean and 95%CI). **e** The same as **d** but for all other regions of England (grouped together)

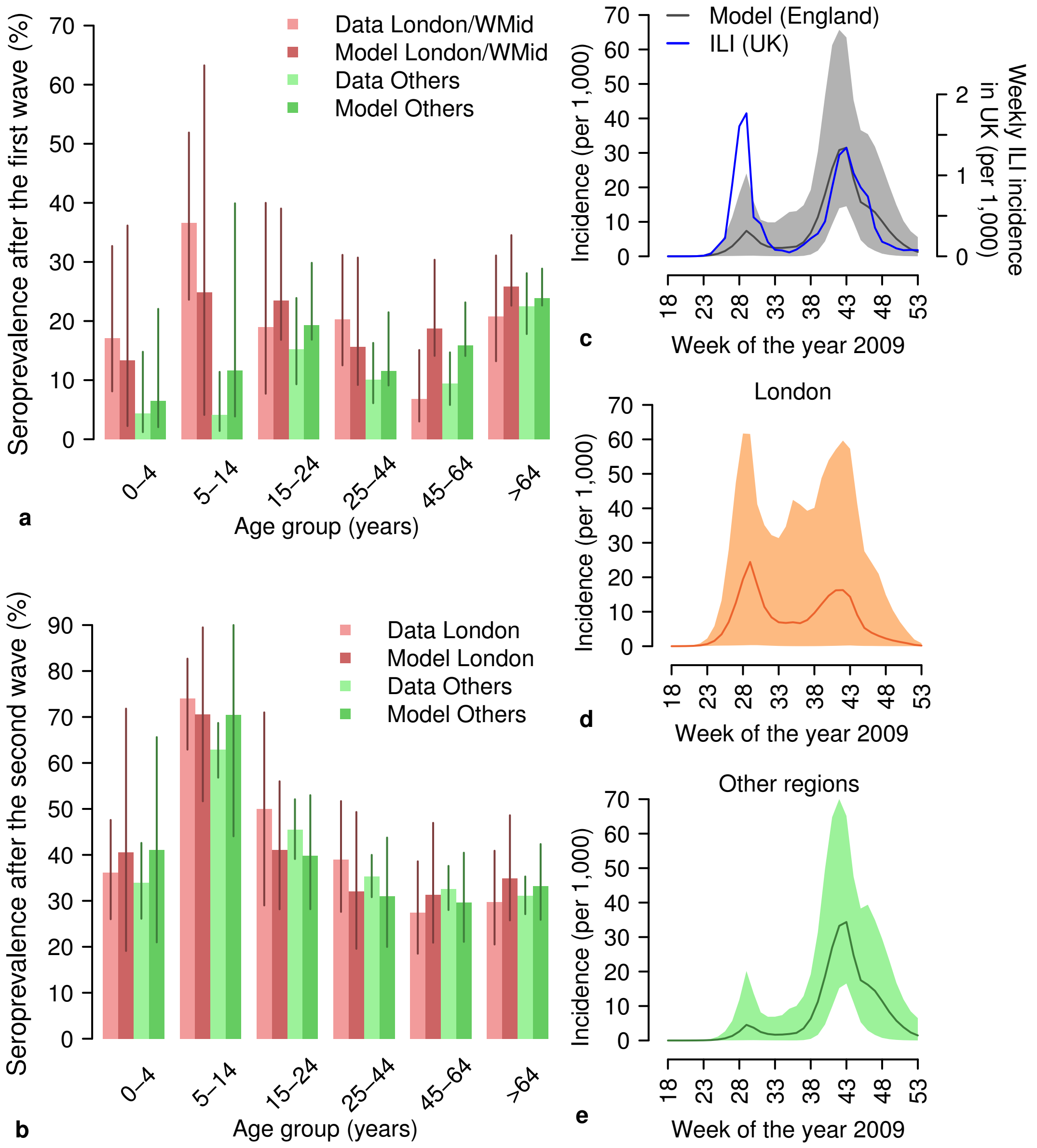

Figure S9: **Spatial spread of 2009 H1N1 influenza pandemic in England as estimated by model M3.** **a** Age-specific seroprevalence (mean, 95% CI) by age group and region as reported in [8, 21] (proportion of serum samples with haemagglutination inhibition titre 1:32 or more) and as estimated by model M3, as of August 2009 (i.e., at the end of the first epidemic wave). Regions are grouped as in the original works [8, 21]; WMid corresponds to West Midlands. **b** as **a**, but as of January 2010 (i.e., at the end of the second wave). **c** Weekly incidence of new reported ILI cases in the UK [2] and weekly incidence of new infections estimated by simulating the calibrated model M3 (mean and 95%CI) for England. **d** Weekly incidence of new infections in London as estimated by model M3 (mean and 95%CI). **e** The same as **d** but for all other regions of England (grouped together)

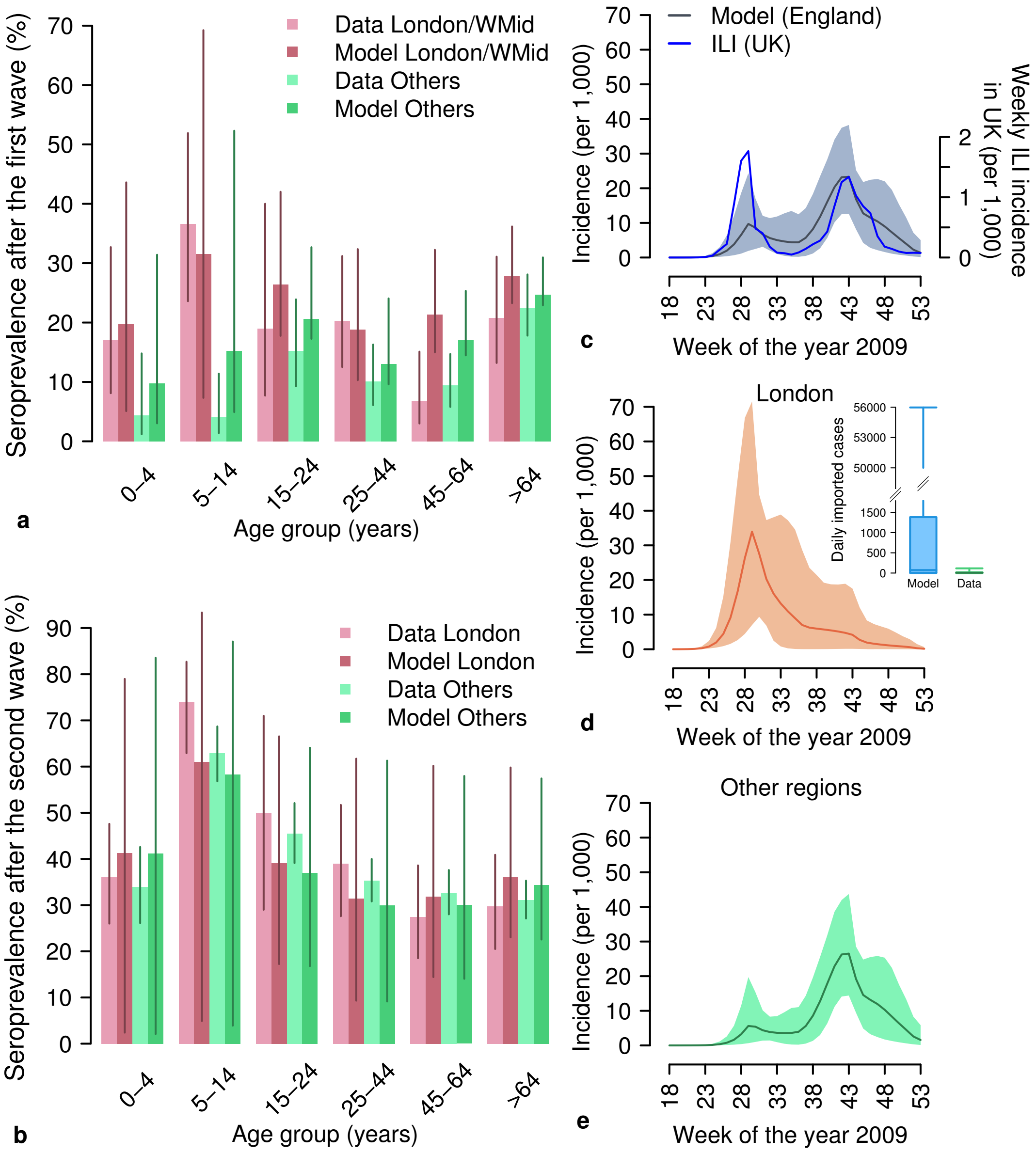

Figure S10: **Spatial spread of 2009 H1N1 influenza pandemic in England as estimated by model M4.** **a** Age-specific seroprevalence (mean, 95% CI) by age group and region as reported in [8, 21] (proportion of serum samples with haemagglutination inhibition titre 1:32 or more) and as estimated by model M4, as of August 2009 (i.e., at the end of the first epidemic wave). Regions are grouped as in the original works [8, 21]; WMid corresponds to West Midlands. **b** as **a**, but as of January 2010 (i.e., at the end of the second wave). **c** Weekly incidence of new reported ILI cases in the UK [2] and weekly incidence of new infections estimated by simulating the calibrated model M4 (mean and 95%CI) for England. **d** Weekly incidence of new infections in London as estimated by model M4 (mean and 95%CI). The inset shows the posterior distribution (median, 50% CI, 95% CI) of the daily number of imported cases as obtained by Model M4 and as reported in the time-series of travel-related cases [16]. **e** The same as **d** but for all other regions of England (grouped together)

3 Supplementary Video S1

Supplementary Video S1 caption. Estimated spatiotemporal dynamics of the pandemic. Spatiotemporal pattern of influenza spread. Left panel shows the simulated median incidence of new weekly influenza cases by cell; right panel shows the simulated cumulative median incidence of weekly cases by cell. Averages computed on 2,000 simulations performed by using model M5. The maps were generated using Grass GIS 6.4.2 (`https://grass.osgeo.org/announces/announce_grass642.html`).

References

[1] Merler S, Ajelli M The role of population heterogeneity and human mobility in the spread of pandemic influenza. Proc R Soc B : 557–565.

[2] Merler S, Ajelli M, Pugliese A, Ferguson NM (2011) Determinants of the spatiotemporal dynamics of the 2009 h1n1 pandemic in europe: Implications for real-time modelling. PLoS Comput Biol 7: e1002205.

[3] Merler S, Ajelli M, Fumanelli L, Vespignani A (2013) Containing the accidental laboratory escape of potential pandemic influenza viruses. BMC Med 11: 252–252.

[4] Fumanelli L, Ajelli M, Manfredi P, Vespignani A, Merler S (2012) Inferring the Structure of Social Contacts from Demographic Data in the Analysis of Infectious Diseases Spread. PLoS Comput Biol 8: e1002673.

[5] Education, Audiovisual and Culture Executive Agency, European Commission (2009). Key Data on Education in Europe. URL `http://ecdc.europa.eu/en/healthtopics/documents/090613_influenza_ah1n1_situation_report_1700hrs.pdf`.

[6] Simini F, Gonzalez MC, Maritan A, Barabasi AL (2012) A universal model for mobility and migration patterns. Nature 484: 96–100.

[7] Department of Transports (2015). National Travel Survey 2013. URL `https://www.gov.uk/government/statistics/national-travel-survey-2013`.

[8] Miller E, Hoschler K, Hardelid P, Stanford E, Andrews N, et al. (2010) Incidence of 2009 pandemic influenza a {H1N1} infection in england: a cross-sectional serological study. Lancet 375: 1100 - 1108.

[9] Ferguson NM, Cummings DAT, Fraser C, Cajka JC, Cooley PC, et al. (2006) Strategies for mitigating an influenza pandemic. Nature 442: 448–452.

[10] Cauchemez S, Donnelly CA, Reed C, Ghani AC, Fraser C, et al. (2009) Household transmission of 2009 pandemic influenza a (h1n1) virus in the united states. N Engl J Med 361: 2619-2627.

[11] Cauchemez S, Valleron AJ, Bolle PY, Flahault A, Ferguson NM (2008) Estimating the impact of school closure on influenza transmission from Sentinel data. Nature 452: 750–754.

[12] Lessler J, Reich NG, Brookmeyer R, Perl TM, Nelson KE, et al. (2009) Incubation periods of acute respiratory viral infections: a systematic review. Lancet Infect Dis 9: 291–300.

[13] Cowling BJ, Fang VJ, Riley S, Malik Peiris JS, Leung GM (2009) Estimation of the serial interval of influenza. Epidemiol 20: 344–347.

[14] Cori A, Valleron A, Carrat F, Tomba GS, Thomas G, et al. (2012) Estimating influenza latency and infectious period durations using viral excretion data. Epidemics 4: 132–138.

[15] Civil Aviation Authority (2009). 2009 Monthly Airport Data. URL `http://www.caa.co.uk/Data-and-analysis/UK-aviation-market/Airports/Datasets/UK-Airport-data/Airport-data-1990-onwards/`.

[16] European Center for Disease Prevention and Control (ECDC) (2009). ECDC Situation report on new influenza A(H1N1) 13 June 2009. URL `http://ecdc.europa.eu/en/healthtopics/documents/090613_influenza_ah1n1_situation_report_1700hrs.pdf`.

[17] Balcan D, Hu H, Goncalves B, Bajardi P, Poletto C, et al. (2009) Seasonal transmission potential and activity peaks of the new influenza A(H1n1): a Monte Carlo likelihood analysis based on human mobility. BMC Med 7: 45.

[18] Khan K, Arino J, Hu W, Raposo P, Sears J, et al. (2009) Spread of a novel influenza A (H1n1) virus via global airline transportation. N Engl J Med 361: 212–214.

[19] Viboud C, Bjørnstad ON, Smith DL, Simonsen L, Miller MA, et al. (2006) Synchrony, waves, and spatial hierarchies in the spread of influenza. Science 312: 447–451.

[20] Balcan D, Colizza V, Gonçalves B, Hu H, Ramasco JJ, et al. (2009) Multiscale mobility networks and the spatial spreading of infectious diseases. Prc Natl Acad Sci USA 106: 21484–21489.

[21] Hardelid P, Andrews N, Hoschler K, Stanford E, Baguelin M (2011) Assessment of baseline age-specific antibody prevalence and incidence of infection to novel influenza a h1n1 2009. Health Technol Assess 14.

[22] Gilks W (2005) Markov Chain Monte Carlo. Wiley Online Library.

[23] Chowell G, Hengartner N, Castillo-Chavez C, Fenimore P, Hyman J (2004) The basic reproductive number of ebola and the effects of public health measures: the cases of congo and uganda. J Theor Biol 229: 119 - 126.

[24] Wallinga J, Lipsitch M (2007) How generation intervals shape the relationship between growth rates and reproductive numbers. Proc R Soc B 274: 599–604.
